# Associations between polymyalgia rheumatica and giant cell arteritis and 12 cardiovascular diseases

**DOI:** 10.1136/heartjnl-2015-308514

**Published:** 2016-01-19

**Authors:** Mar Pujades-Rodriguez, Bram Duyx, Sara L Thomas, Dimitris Stogiannis, Liam Smeeth, Harry Hemingway

**Affiliations:** 1 Clinical Epidemiology Group, Farr Institute of Health Informatics Research, University College London, London, UK; 2 Leeds Institute of Biomedical & Clinical Sciences, MRC Medical Bioinformatics Centre, University of Leeds, Leeds, UK; 3 Faculty of Epidemiology and Population Health, London School of Hygiene and Tropical Medicine, London, UK

## Abstract

**Objectives:**

Evidence of the association of polymyalgia rheumatica (PMR) and giant cell arteritis (GCA) with the full range of cardiovascular diseases (CVDs) is limited. We examined their relationship with the first clinical presentation of the 12 most common CVDs in an unselected population-based cohort of men and women.

**Methods:**

We analysed CArdiovascular disease research using LInked Bespoke studies and Electronic health Records (CALIBER) data, which links primary care and hospital and mortality data in England, from 1997 to 2010. We assembled a cohort of men and women initially free from CVD at baseline and included all patients with PMR and/or GCA (PMR/GCA) diagnosis, matched by age, sex and general practice with up to 10 individuals without PMR/GCA. Random effects Poisson regression analysis was used to study the association between PMR/GCA and the initial presentation of 12 types of CVDs.

**Results:**

The analysis included 9776 patients with PMR only, 1164 with GCA only, 627 with PMR and GCA and 105 504 without either condition. During a median of 3.14 years of follow-up 2787 (24.1%) individuals with PMR/GCA and 21 559 (20.4%) without PMR/GCA developed CVDs. Patients with PMR/GCA had lower rates of unheralded coronary death (3.18 vs 3.61/1000 person-years; adjusted incidence ratio 0.79, 95% CI 0.66 to 0.95), transient ischaemic attack (5.11 vs 5.61/1000 person-years; 0.67, 95% CI 0.54 to 0.84) and coronary and death composite (24.17 vs 25.80/1000 person-years; 0.90, 95% CI 0.82 to 0.98). No associations were observed for other CVDs or cerebrovascular diseases, and in patients with only PMR or GCA. No evidence of interaction by age or sex was found. Estimates decreased with longer PMR/GCA duration and findings were robust to multiple sensitivity analyses.

**Conclusions:**

In this large contemporary population-based cohort the presence of PMR and/or GCA was not associated with an increased risk of CVDs or cerebrovascular diseases regardless of PMR/GCA duration.

## Introduction

Polymyalgia rheumatica (PMR) and giant cell arteritis (GCA) are two chronic inflammatory diseases with similar genetic and acute-phase response patterns that affect predominantly women aged ≥50 years.^1^ ^2^ These diseases are associated with high financial burden related to development of complications, and the number of incident cases is predicted to increase as a result of population aging.^3^

The inflammatory nature of the two conditions and presence of clinical or subclinical arteritis^4–6^ might lead to an increased risk of cardiovascular diseases (CVDs). Findings from the few available studies investigating the association between PMR and/or GCA and CVDs are heterogeneous and conflicting,^7^ with some reporting increased risk and others reporting an absence of association.^8^ ^9^ A recent meta-analysis including six observational studies did not find evidence of increased risk of coronary artery disease in patients with GCA compared with non-GCA.^9^ Most existing studies were conducted in tertiary or secondary care centres, two unpublished studies contributed half of the patients included and most did not adjust for major cardiovascular factors (eg, smoking, blood pressure and/or diabetes). Two large studies analysed primary care records in the UK^8^ ^10^ and found an increased risk of peripheral arterial disease (PAD) and of composite endpoints of coronary and cerebrovascular diseases in patients with PMR^8^ or GCA.^10^ However data were not linked to the mortality register or hospital records and covered a very large time span (1987–1999 and 1990–2010) in which diagnosis practice and clinical management for cardiovascular and chronic inflammatory conditions have considerably evolved. Estimates reported by Tomasson were unadjusted for cardiovascular factors. Furthermore, the study by Hancock excluded patients with another inflammatory rheumatoid disease and patients without smoking information (in adjusted analysis) and definitions of confounders are not available.

Because one in two patients diagnosed with GCA will also develop PMR^11^ and the two diseases are closely related, most published research has included a large proportion of individuals with the two conditions and/or reported combined analyses. However, the strength of the associations with different presentations of CVD might vary for each of the two conditions, and it is important to also assess them separately.

In this longitudinal population-based study of individuals registered in primary healthcare practices with linked information to hospital, registry and mortality data, we examine the association between PMR and GCA and the 12 most common clinical initial presentations of CVD. We also assess whether associations differ according to disease duration, sex, age or presence of other autoimmune diseases.

## Methods

### Study population

All patients with prospectively recorded PMR and/or GCA diagnosis between January 1997 and March 2010 were identified in the CALIBER (CArdiovascular disease research using LInked Bespoke studies and Electronic health Records) dataset.^12^ This dataset links 225 primary care practices in England to hospital and mortality data, and the validity of both risk factor and disease records is supported by results from previous cardiovascular studies.^13–16^

For each patient with PMR/GCA, up to 10 individuals matched on age (±5 years), sex and practice were randomly selected among those who, on their matched inclusion date, had contacted the medical practice within 1 year and had not been diagnosed with the disease (concurrent sampling). At the start of the observation period all patients were ≥18 years old, free of prior CVD and had been registered with their practice for ≥1 year.

### PMR and GCA

Patients with recorded diagnosis of PMR and/or GCA were identified from electronic medical records using Read and ICD-10 codes (see online supplementary table S1). Those with supporting information for PMR or GCA diagnosis (recorded diagnosis in both primary care and hospital; ≥2 corticosteroid prescriptions, ≥1 immunosuppressant drug prescription (see online supplementary table S2) or under the care of a rheumatologist within 6 months) were included in primary analyses (see online supplementary figure S1 and table S3). Patients without supporting information for diagnosis were included in sensitivity analyses. Individuals were initially categorised as having PMR and/or GCA (PMR/GCA group) or not having either condition. Because it is uncertain whether the two conditions are different presentations of the same disease, they were then divided into subgroups of those diagnosed only with PMR, only with GCA or with GCA regardless of previous or later PMR diagnosis during follow-up (all GCA). Patients were further classified into incident and prevalent cases, depending on whether the first recorded diagnosis was made before or after/on study entry.

### Covariates

Baseline covariates considered were: sociodemographic (sex, age and index of multiple deprivation), cardiovascular (diabetes diagnosis, smoking status, body mass index, systolic blood pressure, and total and high-density lipoprotein cholesterol), ≥2 prescriptions in the past year (blood pressure lowering drugs, statins, non-steroidal anti-inflammatory drugs (NSAIDs), immunosuppressant drug, oestrogen oral contraceptives and hormone replacement therapy), diagnosis with another autoimmune diseases (see online supplementary table S4) and year of study entry. As in previous CALIBER analyses,^13–17^ physician diagnoses recorded before or on the entry date were used and the biomarker (eg, blood pressure) measures and smoking status most recently recorded in Clinical Practice Research Datalink (CPRD) up to 1 year before the entry date were used to define baseline covariates. Definitions can be found at https://www.caliberresearch.org/portal/.

### Endpoints

The primary study endpoints were the initial presentation of 12 fatal and non-fatal cardiac and arterial vascular diseases of heterogeneous pathophysiology identified across the four linked data sources in CALIBER: the CPRD^18^; the Myocardial Ischaemia National Audit Project disease registry^19^; Hospital Episodes Statistics; and the national death registry. CVD presentations studied were: coronary diseases (stable angina, unstable angina, myocardial infarction and unheralded coronary death), cardiac diseases (heart failure and cardiac arrest), cerebrovascular diseases (transient ischaemic attack (TIA), ischaemic stroke, subarachnoid haemorrhage and intracerebral haemorrhage), PAD and abdominal aortic aneurysm. Secondary endpoints were two composite outcomes, coronary and CVD death (including myocardial infarction, stable angina, coronary heart diseases not otherwise specified and cardiovascular death); and fatal and non-fatal CVD (additionally including TIA, PAD, non-fatal ischaemic or haemorrhagic stroke and heart failure); and first event of each CVD type (ie, regardless of other earlier CVD presentations during follow-up). Diagnosis codes can be found at http://www.caliberresearch.org/portal/.

### Study follow-up period

For individuals with PMR/GCA the observation period began on the date on which the patient fulfilled all the study inclusion criteria, or the date of first recorded diagnosis for patients with incident PMR/GCA (see online supplementary figure S2). For individuals without PMR/GCA, it started on the entry date of the matched individual with PMR/GCA. For all patients the study ended on the date of cardiovascular endpoint diagnosis, death, practice deregistration, last practice data collection date or diagnosis of PMR/GCA (for patients who contributed follow-up to both the PMR/GCA and non-PMR/GCA groups).

### Statistical analysis

Incidence rates of events per 1000 person-years of follow-up with 95% CIs were calculated in patients with and without PMR/GCA, separately for individuals with prevalent or incident PMR/GCA, and for patients exclusively diagnosed with PMR or GCA. Associations between PMR/GCA and every study endpoint were assessed with random effects Poisson regression accounting for clustering between practices. Missing values of covariates were multiply imputed (see online supplementary text S1). Models were initially adjusted for age and sex, then additionally for cardiovascular factors. To explore possible pathways or mediators of any increase in risk, estimates were further adjusted for: (i) lipid and blood pressure lowering medication; (ii) diagnosis of another autoimmune disease; and (iii) anti-inflammatory and/or immunosuppressant drugs.

In secondary analyses: (i) evidence of interactions with sex and age were assessed using Wald tests for interaction; (ii) the relationships with prevalent and incident PMR/GCA were studied; (iii) associations were assessed separately for patients who had only one of the two diseases and for those who had GCA regardless of PMR diagnosis. Furthermore, to examine the association between disease duration and CVD, the follow-up of patients with incident PMR/GCA was split into duration periods (0–1, 2–4 and ≥5 years). Patients with prevalent disease who had a first diagnosis recorded ≥5 years before study entry were included in the ≥5 year period group. Those with prevalent disease of shorter duration (<5 years) were excluded from this analysis because of uncertainty about exact disease duration.

Sensitivity analyses comprised: (i) exclusion of patients with another autoimmune disease; (ii) restriction to patients with ≥6 months of follow-up; and (iii) exclusion of 2 years of follow-up prior to PMR/GCA diagnosis for patients who contributed to the exposed and unexposed groups (to account for delays in diagnosis); (iv) inclusion of patients without supporting information for PMR/GCA diagnosis; (v) exclusion of patients of <50 years of age; (vi) inclusion of first cardiovascular events irrespective of other earlier CVD presentations; (vii) comparison of estimates in the periods before and after the introduction of the pay for performance scheme in England (Quality of Outcomes Framework); and (viii) restriction to patients with evidence of active disease (baseline c-reactive protein (CRP) >3.0 mg/mL or elevated erythrocyte sedimentation rate (ESR)).

Statistical analyses were done with Stata (V.13.1).

### Ethical considerations

Approval was granted by the Independent Scientific Advisory Committee of the Medicines and Healthcare Products Regulatory Agency and the MINAP Academic Group. We registered the protocol at clinicaltrials.gov (NCT02062021).

## Results

### Baseline patient characteristics

**Figure 1:**
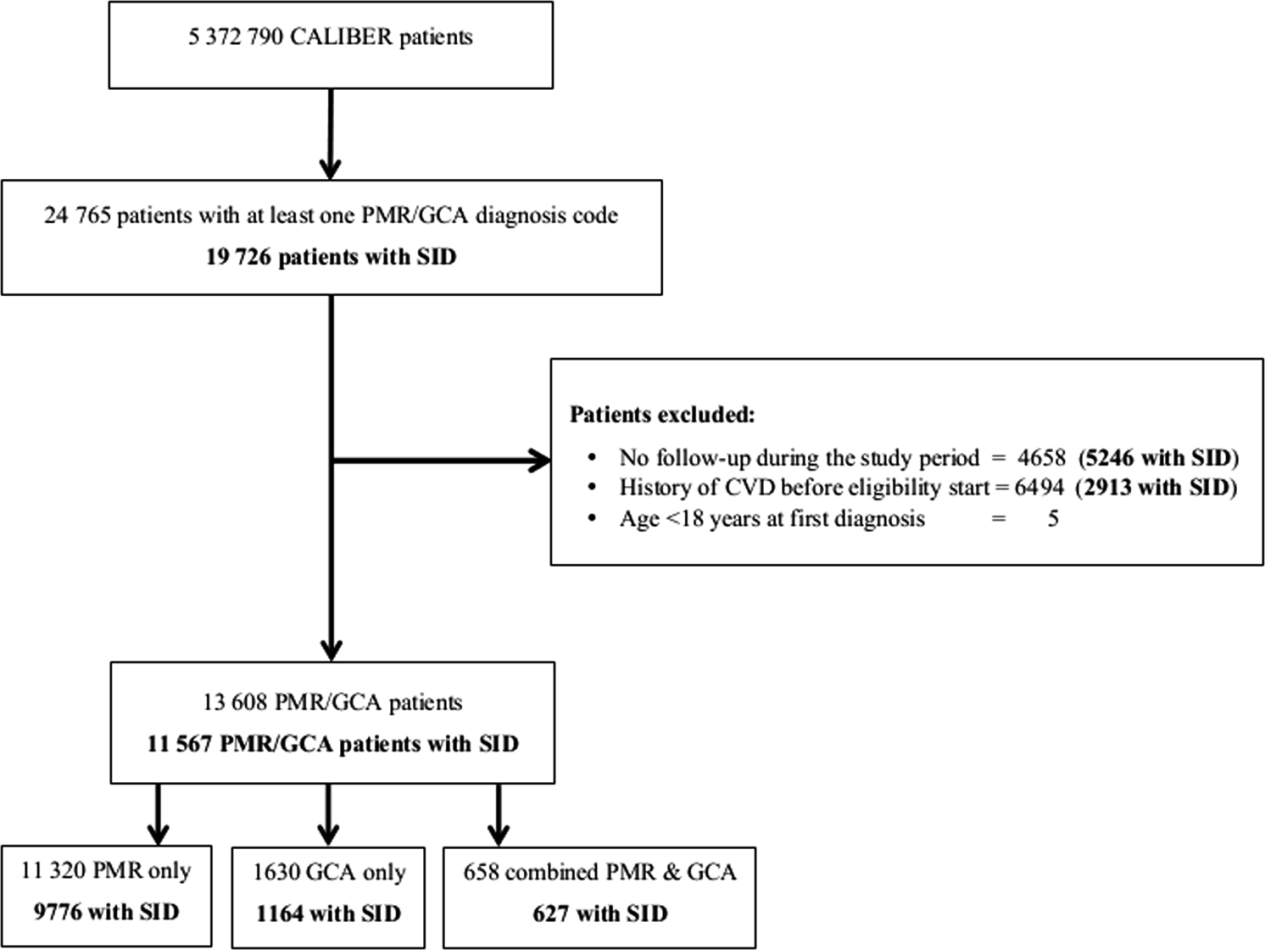
Study flow diagram. CALIBER, CArdiovascular disease research using LInked Bespoke studies and Electronic health Records; CVD, cardiovascular disease; GCA, giant cell arteritis; PMR, polymyalgia rheumatica; SID, supporting information for disease diagnosis.

In total 11 567 patients with PMR/GCA and supporting diagnosis information and 105 504 non-PMR/GCA patients free of CVD were identified (figure 1). Of patients with PMR/GCA, 9776 (84.5%) had only PMR, 1164 (10.1%) only GCA and 627 (5.4%) were diagnosed with both PMR and GCA. A total of 1848 contributed follow-up time to both the PMR/GCA and non-PMR/GCA cohorts (see online supplementary figure S2). Of the 11 567 patients with PMR/GCA, 8238 (71.2%) had incident disease, and of the 3329 patients with prevalent disease, 26.4% had been diagnosed ≥5 years before study entry. Overall, median duration since practice registration was 13.5 years (IQR 6.1–25.5). Baseline median age was 70 years (IQR 63–77), and 72.3% of patients were women (see table 1 and online supplementary table S5). Patients contributed 473 029 person-years of follow-up, median of 3.13 years (IQR 1.3–6.0). In the year before entry 48.1% of PMR/GCA patients had received ≥2 prescriptions of oral corticosteroids (36.5% of incident and 77.0% of prevalent cases), 30.0% NSAIDs and 1.4% immunosuppressant drugs. Furthermore, evidence of long-term use of corticosteroids (Readcode 14P8.00) was found for 97.9% of patients with PMR and/or GCA during follow-up. Diagnosis with another autoimmune disease was recorded in 11.7% of PMR/GCA patients and in 6.7% of non-PMR/GCA patients. Mean systolic blood pressure was similar in individuals with and without PMR/GCA but higher proportions of PMR/GCA patients received blood pressure lowering medication in the year before study entry (43.0% vs 36.7% in non-PMR/GCA patients). The proportion of patients diagnosed with diabetes was higher among individuals with GCA only (9.7% compared with 6.9% in the PMR group and 6.2% in the non-PMR/GCA group).

**Table 1:** Characteristics* of individuals with PMR, GCA and without either condition

	Patients with PMR only (N=9776)	Patients with GCA only (N=1164)	Patients without PMR or GCA (N=105 504)
*Sociodemographic factors*			
Age, years, median (IQR)	73 (65–79)	72 (64–79)	70 (62–77)
Women, n (%)	7042 (72.0)	845 (72.6)	76 286 (72.3)
Index of multiple deprivation, quintiles, n (%)			
1 (least deprived)	1977 (20.3)	211 (18.2)	21 062 (20.0)
5 (most deprived)	1892 (19.4)	262 (22.6)	20 763 (19.8)
Ethnicity, n (%)			
White	6683 (97.9)	844 (96.6)	62 766 (97.3)
Asian	77 (1.1)	14 (1.6)	768 (1.2)
Afro-Caribbean	21 (0.3)	6 (0.7)	448 (0.7)
Other	47 (0.7)	10 (1.1)	506 (0.8)
Duration of registration, years, median (IQR)	14.3 (6.7–26.9)	13.9 (6.4–26.1)	13.4 (6.0–25.3)
Consultation rate in previous year	10 (6–15)	10 (6–16)	5 (2–9)
*Autoimmune diseases and inflammation*			
Other autoimmune disease, n (%)	1008 (10.3)	130 (11.2)	7116 (6.7)
*Cardiovascular risk factors*			
Smoking, n (%)			
Current	794 (9.0)	171 (16.6)	10 397 (11.4)
Former	2416 (27.3)	273 (26.5)	22 459 (24.6)
Never	5636 (63.7)	585 (56.9)	58 584 (64.1)
Diabetes	671 (6.9)	81 (9.7)	6526 (6.2)
Systolic blood pressure, mm Hg, mean (SD)	144 (18.4)	143 (19.7)	143 (19.0)
Body mass index, kg/m^2^, mean (SD)	26.9 (5.1)	26.8 (5.6)	26.7 (5.1)
Total cholesterol, mmol/L, mean (SD)	5.6 (1.2)	5.6 (1.2)	5.6 (1.1)
HDL cholesterol, mmol/L, mean (SD)	1.6 (0.5)	1.5 (0.5)	1.5 (0.5)
Serum creatinine, mg/L, mean (SD)	87.5 (24.3)	87.2 (21.8)	88.2 (24.3)
*Medication use in previous year*			
Immunosuppressant drugs, n (%)	143 (1.5)	–	593 (0.6)
Non-steroidal anti-inflammatory drugs, n (%)	3080 (31.5)	248 (21.3)	14 240 (13.5)
Oral corticosteroids, n (%)	4713 (48.2)	503 (43.2)	2476 (2.4)
Antiplatelet therapy, n (%)	843 (8.6)	123 (10.6)	8787 (8.3)
Blood pressure lowering medication, n (%)	4253 (43.5)	477 (41.0)	38 704 (36.7)
Statins, n (%)	978 (10.0)	102 (8.8)	9230 (8.8)

*Missing data (%): index of multiple deprivation, 0.4%; ethnic group, 37.9%; BMI, 41.8%; systolic blood pressure, 13.0%; smoking, 13.0%; total cholesterol, 61.2%; HDL cholesterol, 71.0%; serum creatinine, 47.6%.BMI, body mass index; GCA, giant cell arteritis; HDL, high-density lipoprotein; IRR, incidence rate ratio; PMR, polymyalgia rheumatica.

### Association between PMR/GCA and the 12 CVDs

**Figure 2:**
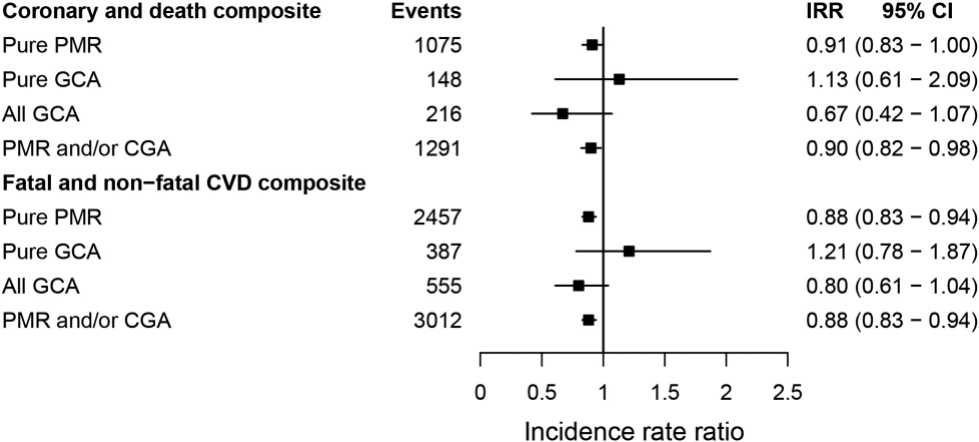
Adjusted incidence rate ratios for the association between polymyalgia rheumatica and giant cell arteritis, and the initial presentation of coronary disease and CVD. The coronary and CVD death composite endpoint includes: stable angina, myocardial infarction, coronary heart diseases not otherwise specified and any cardiovascular death. The fatal and non-fatal CVD composite endpoint additionally includes: transient ischaemic attack, peripheral arterial disease, and non-fatal heart failure, ischaemic or haemorrhagic stroke. CVD, cardiovascular disease; giant cell arteritis; IRR, incidence rate ratios adjusted for index of multiple deprivation, smoking status, systolic blood pressure, total and high-density lipoprotein cholesterol, body mass index and diabetes; n, number of events; PMR, polymyalgia rheumatic.

**Figure 3:**
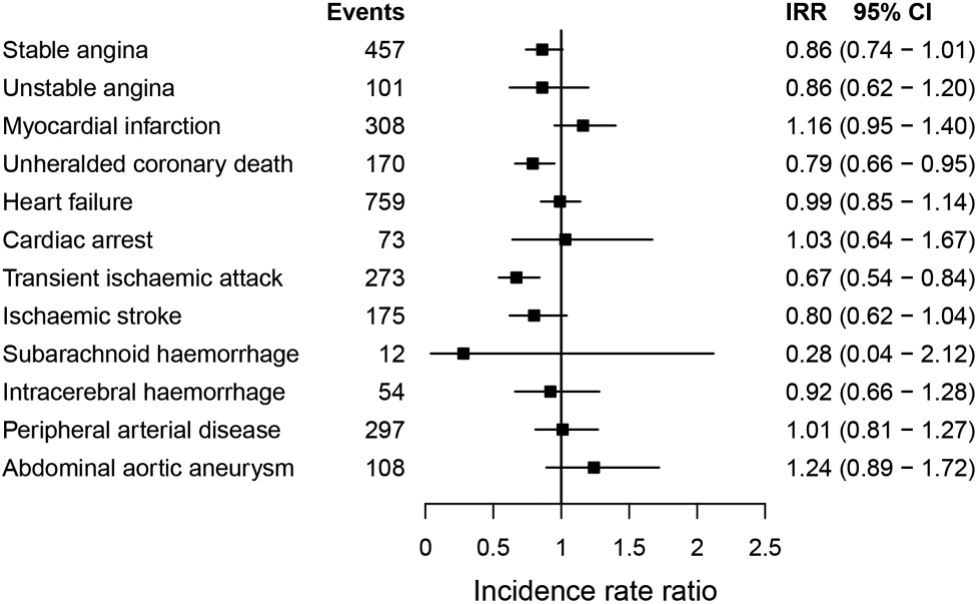
Adjusted incidence rate ratios for the association between polymyalgia rheumatica and/or giant cell arteritis, and the initial presentation of 12 cardiovascular diseases. Because of the small number of events in specific clusters, estimates for unheralded coronary death and intracerebral haemorrhage were not adjusted for body mass index, systolic blood pressure, and total and high-density lipoprotein cholesterol. IRR, incidence rate ratios adjusted for index of multiple deprivation, smoking status, systolic blood pressure, total and high-density lipoprotein cholesterol, body mass index and diabetes; n, number of events.

During follow-up, 2272 (23.2%) patients with PMR only, 348 (29.9%) with GCA only, 167 (26.6%) with both conditions and 21 559 (20.4%) without either disease experienced one of the 12 CVDs. The incidence of fatal and non-fatal CVDs was slightly lower in patients with than without PMR/GCA (adjusted IRR=0.88, 95% CI 0.83 to 0.94; table 2 and figure 2). Patients with PMR and/or GCA had lower incidence rates of unheralded coronary death (IRR=0.79, 95% CI 0.66 to 0.95), and TIA (IRR=0.67, 95% CI 0.54 to 0.84) than non-PMR/GCA patients (see figure 3; online supplementary tables S6 and S7). No evidence of association was found between PMR/GCA and any of the other cardiac, cerebrovascular or vascular endpoints. Additional adjustment for baseline medication, diagnosis of other autoimmune diseases (see online supplementary figures S3 and S4) or year of entry (see online supplementary figure S5) did not alter the estimates. No evidence of effect modification by age or sex was found (see online supplementary figures S6 and S7).

**Table 2:** Crude incidence rates of 12 CVDs with 95% CIs in individuals with and without PMR and GCA

	Coronary and CVD death composite		Fatal and non-fatal cardiovascular composite	
	No. Events	Rate per 1000 PY (95% CI)	No. events	Rate per 1000 PY (95% CI)
PMR only	1075	23.95 (22.56 to 25.43)	2457	54.74 (52.62 to 56.95)
GCA only	148	30.44 (25.91 to 35.76)	387	79.60 (72.05 to 87.94)
All GCA	216	25.32 (22.16 to 28.93)	555	65.06 (59.86 to 70.70)
PMR and/or GCA	1291	24.17 (22.89 to 25.52)	3012	56.39 (54.41 to 58.44)
Free of PMR and GCA	10 826	25.80 (25.32 to 26.29)	24 372	58.08 (57.36 to 58.82)

*Notes*: non-disease estimates are obtained in up to 10 randomly selected patients without PMR or GCA matched for sex, age, medical practice and index date; PY rates are unadjusted; PMR and GCA estimates are obtained among patients diagnosed with these diseases who had supporting information for disease diagnosis. The coronary and CVD death composite endpoint includes: stable angina, myocardial infarction, coronary heart diseases not otherwise specified and any cardiovascular death. The fatal and non-fatal CVD composite endpoint additionally includes: transient ischaemic attack, peripheral arterial disease and non-fatal heart failure and ischaemic or haemorrhagic stroke.

CVD, cardiovascular disease; GCA, giant cell arteritis; PMR, polymyalgia rheumatica; PY, patient-years of follow-up.

**Figure 4:**
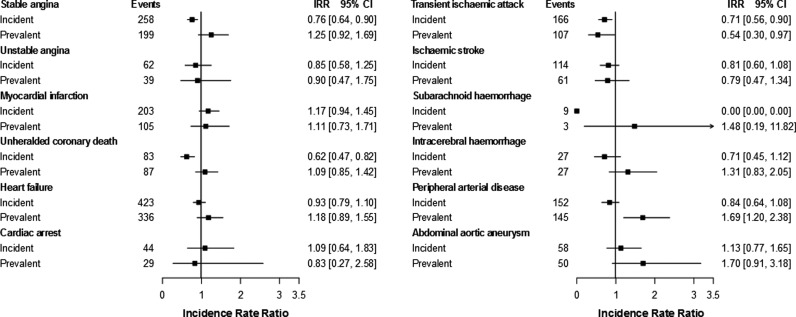
Adjusted incidence rate ratios of the initial presentation of 12 cardiovascular diseases for incident and prevalent polymyalgia rheumatica and/or giant cell arteritis (vs no disease). Because of the small number of events in specific clusters, estimates for unheralded coronary death and intracerebral haemorrhage were not adjusted for body mass index, systolic blood pressure, and total and high-density lipoprotein cholesterol. IRR, incidence rate ratios for patients with polymyalgia rheumatica and/or giant cell arteritis adjusted for sex, age, index of multiple deprivation, smoking status, systolic blood pressure, total and high-density lipoprotein cholesterol, body mass index and diabetes.

Lower incidence ratios were generally observed for patients with incident than prevalent PMR/GCA (see figure 4 and online supplementary table S7), and non-overlapping 95% CIs were found for stable angina (IRR=0.76, 95% CI 0.64 to 0.90 vs 1.25, 95% CI 0.92 to 1.69), unheralded coronary death (IRR=0.62, 95% CI 0.47 to 0.82 vs 1.09, 95% CI 0.85 to 1.42) and PAD (IRR=0.84, 95% CI 0.64 to 1.08 vs 1.69, 95% CI 1.20 to 2.38). Among individuals with PMR/GCA, the lowest rates of CVD were generally observed after 5 years of disease duration (see online supplementary figure S8); with no overlapping 95% CIs found for stable angina (adjusted IRR=0.73, 95% CI 0.65 to 0.81 for 2–4 years; and IRR=0.43, 95% CI 0.30 to 0.62 for ≥5 years compared with ≤1 year duration) and for heart failure (IRR=0.83, 95% CI 0.77 to 0.90 and IRR=0.61, 95% CI 0.49 to 0.76, respectively).

Incidence of CVD was higher in the GCA than in the pure PMR group (70.60 vs 54.74/1000 person-years), greater differences being observed for heart failure, PAD and stable angina. The small number of CVD events in the subgroup of patients with GCA limited the power to examine differences in rate ratios between patients with PMR and GCA for some CVDs. However, no evidence of increased risk of CVD was observed for those with a higher number of events (see online supplementary table S8) and for composites endpoints (figure 2).

### Results from sensitivity analyses

Exclusion of individuals diagnosed with another autoimmune disease or patients aged <50 years, inclusion of patients with >6 months of follow-up and censoring of follow-up 2 years before the diagnosis of PMR/GCA in patients who contributed to both, PMR/GCA and non-PMR/GCA groups, did not alter the results (see online supplementary table S8 and figure S10). Patterns of association remained unchanged when PMR/GCA individuals were included irrespective of whether disease supporting information was recorded (see online supplementary figure S11). Analyses comparing estimates before and after the introduction of the pay-for-performance system of CPRD, those of first presentation of CVD (ie, regardless of other earlier CVD presentations) and those restricted to patients with evidence of active disease also showed similar patterns of associations (see online supplementary figures S12 and S13 and table S10).

## Discussion

This is to date the largest population-based longitudinal study comparing the initial presentation of a wide range of symptomatic CVDs in patients with and without PMR and/or GCA using contemporary data (1998–2010). The large sample size, the longitudinal design and the information available allowed examination of associations in individuals with prevalent and incident PMR/GCA, pure and concomitant PMR and GCA, different duration of disease and adjustment for sociodemographic and cardiovascular factors. In contrast to a previous CALIBER study conducted in the same population in which we reported increased rates of the initial presentation of several CVDs in patients with rheumatoid disease (awaiting publication), no evidence of increased risk of any of the 12 CVD presentations investigated was found in the presence of PMR/GCA.

This study has several strengths. The longitudinal population-based design provided evidence of temporality and enabled investigation of associations with duration of PMR/GCA since diagnosis. The large sample size allowed conducting separate analyses for patients with incident and prevalent PMR/GCA and pure and concomitant PMR and GCA, and investigation of sex and age interactions. The time period covered (1997–2010) provided epidemiological evidence from a population diagnosed and treated in recent years. The analysis of linked electronic health records spanning primary, hospital and disease registry data improved disease and cardiovascular endpoint ascertainment,^20^ and allowed determination of outcomes independently of PMR/GCA status and comparison with a non-disease random sample drawn from the same population. Furthermore, the matching strategy minimised the possibility of healthy study bias related to healthcare service usage and the old age of the PMR/GCA cohort.

Study limitations include the possibility of PMR/GCA misclassification (eg, patients with atypical or mild disease might be less likely to be diagnosed). In our study, PMR/GCA was defined based on recorded physician diagnosis in primary care and hospitals. Information on American College of Rheumatology classification criteria or histology was not available in the dataset for diagnosis confirmation. However, to reduce the likelihood of PMR/GCA misclassification in patients >60 years of age with incident seronegative rheumatoid arthritis or malignancies,^21^ the primary analysis was restricted to patients who had supporting information for diagnosis (eg, 24% of PMR/GCA patients had diagnoses recorded in both primary and hospital care, and 98% received prolonged corticosteroid therapy). Furthermore, the majority of patients with PMR/GCA were women and the median age was 73 years, all characteristics typical of patients with PMR and/or GCA, and exclusion of patients with other systemic diseases (eg, rheumatoid arthritis) did not affect the estimates. Concerning PMR versus GCA diagnosis, 35% of patients with GCA also had PMR and the number of patients with PMR was eight times higher than that of those with GCA, which is also consistent with previous reports.^2^ Furthermore, adjustment for disease activity was not possible (baseline ESR and CRP were available for 8% and 4% of non-PMR/GCA patients, respectively); but analyses restricted to patients with active PMR/GCA did not change the conclusions.

There are several possible explanations for the absence of increased risk of CVD observed in patients with PMR/GCA regardless of disease duration. PMR/GCA affects an old population who might die before a detrimental effect of chronic systemic inflammation on the arteries becomes symptomatic.^9^ Furthermore, the chronic use of corticosteroids (98% of PMR/GCA patients in our cohort) might reduce the progression rate of atherosclerosis through control of systemic inflammation.^22^ This is supported by our finding of decreasing ratios of most types of CVDs among patients with longer disease duration. Finally, unlike most previous studies that were conducted in tertiary care centres, our cohort included all patients treated at primary care and hospital level, making results more complete and representative of the entire PMR/GCA population. Indeed, PMR is primarily diagnosed and treated in primary care, and studies conducted exclusively among patients managed at secondary or tertiary care levels are likely to include high proportions of individuals with more severe disease or with atypical presentation.^7^

Discordance with previous studies that found evidence of increased risk of CVD in patients with PMR/GCA could be related to their inclusion of patients with a history of CVDs and/or primarily diagnosed and treated in tertiary health facilities, and to better^23^ control of cardiovascular factors and early start of anti-inflammatory medication, which could reduce and control systemic inflammation^24^ ^25^ as achieved in current clinical practice for patients with chronic inflammatory conditions. In our study, over 40% of patients with PMR/GCA received blood pressure lowering medication, 10% statins and 9% antiplatelet therapy.

In conclusion, regardless of disease duration, diagnosis of pure or concomitant PMR and GCA was not associated with increased risk of CVDs in a large, contemporary population-based sample suggesting that, within the framework of current clinical practice, these diseases do not represent an important risk factor for CVD.

**Key messages**

**What is already known on this subject?**

- The chronic inflammatory nature of polymyalgia rheumatica (PMR) and giant cell arteritis (GCA) and the presence of clinical or subclinical arteritis might lead to an increased risk of cardiovascular diseases (CVDs). However, findings from the few available studies investigating these associations are heterogeneous and inconsistent.

**What might this study add?**

- In this large, contemporary longitudinal population-based study of patients treated at primary and hospital levels, we examined the associations between prevalent and incident PMR/GCA (PMR/GCA diagnosed before or after/on the date of study entry, respectively) and pure (ie, diagnosis with PMR or GCA only) and concomitant PMR and GCA, and the 12 most common clinical presentations of CVDs. Regardless of PMR/GCA disease duration, presence of pure or concomitant PMR and GCA was not associated with an increased risk of CVD. No evidence of interaction by sex or age was found.

**How might this impact on clinical practice?**

- The study findings suggest that, under current clinical practice targeted to patients with PMR and/or GCA, including early and long-term administration of anti-inflammatory and immunosuppressant drugs to control chronic systemic inflammation associated with PMR/GCA, and management of cardiovascular factors with primary prevention, cardiovascular patient risk is similar to the risk of patients without PMR/GCA.

## Supplementary Material

Web supplement
