# Supplementary material for: Associations between polymyalgia rheumatica and giant cell arteritis and 12 cardiovascular diseases
Source: Heart. 2016 Jan 19;102(5):383–9. doi: 10.1136/heartjnl-2015-308514 (PMC4789702; doi:10.1136/heartjnl-2015-308514)
Supplement: Web supplement [file heartjnl-2015-308514-s1.pdf]

SUPPLEMENTARY MATERIAL

TEXT

Text S1. Multiple imputation

TABLES

Table S1. Read and ICD 10 diagnosis codes for polymyalgia rheumatica and giant cell arteritis

Table S2. List of drugs included as immunosuppressant drugs or biologic therapy

Table S3. Frequency of recorded supporting information suggestive of polymyalgia rheumatica or giant cell arteritis

Table S4. List of diseases considered in the 'other autoimmune disease' covariateTable S5. Characteristics of individuals with and without polymyalgia rheumatic and giant cell arteritis

Table S6. Crude incidence rates of twelve cardiovascular diseases with 95% confidence intervals in individuals with and without polymyalgia rheumatica and giant cell arteritis

Table S7. Crude incidence rates of twelve cardiovascular diseases with 95% confidence intervals in individuals with incident and prevalent polymyalgia rheumatica and/or giant cell arteritis

Table S8. Adjusted incidence rate ratios for the association between pure and concomitant polymyalgia rheumatica and giant cell arteritis, and the initial presentation of cardiovascular diseases

Table S9. Adjusted incidence rate ratios for the association between anti-inflammatory medication use and twelve cardiovascular diseases

Table S10. Adjusted incidence rate ratios for the association between pure and concomitant PMR and GCA, and the initial presentation of cardiovascular diseases, restricted to patients who had evidence of active disease

Table S11. Description of diagnosis codes for patients who were diagnosed with both polymyalgia rheumatica and giant cell arteritisTable S12. Adjusted incidence rate ratios for the association between pure and concomitant PMR and GCA, and the initial presentation of cardiovascular diseases, restricted to patients who had ≥1 diagnosis code recorded after 1 year of the initial diagnosis

FIGURES

Figure S1. Electronic health record phenotyping algorithm for polymyalgia rheumatica and giant cell arteritis

Figure S2. Polymyalgia rheumatica and giant cell arteritis and study definitions

Figure S3. Sequential adjustment of incidence rate ratios for the association between polymyalgia rheumatica and/or giant cell arteritis, and the initial presentation of twelve cardiovascular diseases

Figure S4. Adjusted incidence rate ratios for the association between polymyalgia rheumatica and/or giant cell arteritis, and the initial presentation of twelve cardiovascular diseases, additionally adjusted for the effect of anti-inflammatory medication

Figure S5. Adjusted incidence rate ratios for the association between polymyalgia rheumatica and/or giant cell arteritis, and the initial presentation of twelve cardiovascular diseases further adjusted of year of entry

Figure S6. Adjusted incidence rate ratios for the association between polymyalgia rheumatica and/or giant cell arteritis, and the initial presentation of twelve cardiovascular diseases stratified by age group

Figure S7. Adjusted incidence rate ratios for the association between polymyalgia rheumatica and/or giant cell arteritis, and the initial presentation of twelve cardiovascular diseases in men and women

Figure S8. Adjusted incidence rate ratios for the initial presentation of cardiovascular diseases stratified by disease duration among patients with polymyalgia rheumatica and/or giant cell arteritis (vs. no disease)

Figure S9. Adjusted incidence rate ratios of the initial presentation of twelve cardiovascular diseases for individuals with and without polymyalgia rheumatica, giant cell arteritis, or giant cell arteritis regardless of concomitant diagnosis with polymyalgia rheumatica (vs. no disease)

Figure S10. Adjusted incidence rate ratios for the association between polymyalgia rheumatica and/or giant cell arteritis, and the initial presentation of twelve cardiovascular diseases from sensitivity analyses

Figure S11. Comparison of adjusted incidence rate ratios for the association between polymyalgia rheumatica and/or giant cell arteritis, and the initial presentation of twelve cardiovascular diseases according to disease definition

Figure S12. Adjusted incidence rate ratios for the association between polymyalgia rheumatica and/or giant cell arteritis, and the initial presentation of twelve cardiovascular diseases before and after the introduction of pay for performance (April 2004)

Figure S13. Adjusted incidence rate ratios for the association between polymyalgia rheumatica and/or giant cell arteritis, and the initial and first presentation of twelve cardiovascular diseases

TEXT

Text S1. Multiple imputation

Risk factor data appeared to be missing at random after adjusting for major confounders (e.g. age, sex, diabetes, BMI and blood pressure). Hence, multiple imputation was implemented using the *M*[1] algorithm in the statistical package Stata 13.1, to replace missing values in exposure and risk factor variables. Imputation models were estimated separately for men and women and included:

i) all the baseline covariates used in the main analysis (age, quadratic age, diabetes, smoking, systolic blood pressure, total cholesterol, HDL cholesterol, index of multiple deprivation; diagnosis of another autoimmune disease);

ii) prior (between 1 and 4 years before study entry) and post (between 0 and 1 year after study entry) averages of continuous covariates in the main analysis;

iii) baseline, prior and post average measurements of covariates not considered in the main analysis (diastolic blood pressure, alcohol intake, white blood cell count, haemoglobin, creatinine);

iv) polymyalgia rheumatica and giant cell arteritis status (no, incident, prevalent);

v) baseline medications (nonsteroidal anti-inflammatory drugs, immunosuppressant drugs or biologic therapy, oral corticosteroids, antiplatelet medication, statins, blood pressure lowering medication, low-dose aspirin, loop diuretics, oral contraceptives and hormone replacement therapy);

vi) coexisting medical conditions (hypertension, history of depression, cancer, renal disease, liver disease, other autoimmune disorders and chronic obstructive pulmonary disease);

vii) the Nelson-Aalen hazard and the event status for each of the 12 endpoint analysed[2;]

viii) other baseline information: ethnicity, month of registration, abnormal erythrocyte sedimentation rate (binary variable), and number of consultations within 1 year before date of entry.

Non-normally distributed variables were log-transformed for imputation and back-exponentiated to their original scale for analysis. Ten multiply imputed datasets were generated, and Poisson models were fitted to each dataset. Coefficients were combined using Rubin's rules. The Kolmogorov-Smirnov test was used to compare the distribution of observed versus imputed log-transformed covariates.

Reference list

(1) Royston P, White IR, Multiple imputation by chained equations (MICE): Implementations in STATA, Journal of Statistical Software, 2011; **45**(4): 1-20.
(2) van Buuren S. Muliple imputation of discrete and continuous data by fully conditional specification. Stat Methods Med Res. 2007; 16: 219-242.

TABLES

Table S1. Read and ICD 10 diagnosis codes for polymyalgia rheumatica and giant cell arteritis

**Read codes**	**Polymyalgia rheumatica**
N20..00	Polymyalgia rheumatica
N20..00	Polymyalgia
N200.00	Giant cell arteritis with polymyalgia rheumatica
	**Giant cell arteritis**
G755100	Temporal arteritis
G755000	Cranial arteritis
G755.00	Giant cell arteritis
N200.00	Giant cell arteritis with polymyalgia rheumatica
Nyu4100	[X]Other giant cell arteritis
G755z00	Giant cell arteritis NOS
**ICD 10 codes**	
	**Polymyalgia rheumatica**
M353	Polymyalgia rheumatica
M315	Giant cell arteritis with polymyalgia rheumatica
	**Giant cell arteritis**
M316	Other giant cell arteritis

Table S2. List of drugs included as immunosuppressant drugs or biologic therapy

Azathioprine
Cyclosporin
Cyclophosphamide
Hydroxychloroquine sulphate
Leflunomide
Methotrexate
Rituximab*

*Only for patients with giant cell arteritis

Table S3. Frequency of recorded supporting information suggestive of polymyalgia rheumatica or giant cell arteritis

	**Patients with PMR/GCA** N=11,567
**Source of diagnosis**	
Diagnosis in CPRD (primary care)	11,420 (98.7)
Diagnosis in HES (hospital care)	3,189 (27.6)
Diagnosis in CPRD and HES	3,099 (26.8)
**Receiving care by a rheumatologist within 6 months of a recorded diagnosis**	1,453 (12.6)
**Referred to a rheumatologist within 6 months before diagnosis**	565 (4.9)
**Medication prescription within 6 months of a recorded diagnosis**	390 (33.6)
**Long term use of oral corticosteroids**	11,326 (97.9)

Note: CPRD, the Clinical Practice Research Datalink; GCA, giant cell arteritis; HES, Hospital Episode Statistics; PMR, polymyalgia rheumatica. Overall, 85% of patients diagnosed with PMR and/or GCA had supporting information for diagnosis.

Table S4. List of diseases considered in the 'other autoimmune disease' covariate

Ankylosis spondylitis
Autoimmune bullous disease
Autoimmune uveitis
Behcet's disease
Dermato-polymyositis
Henoch-Schönlein purpura
Multiple sclerosis
Polyarteritis nodosa
Primary biliary cirrhosis
Psoriasis
Rheumatoid arthritis
Sjögren's syndrome
Systemic lupus erythematosus
Systemic sclerosis
Wegener's granulomatosis

Table S5. Characteristics* of individuals with and without polymyalgia rheumatica and giant cell arteritis

	Patients with PMR and/or GCA (N=11,567)	Patients without either PMR or GCA (N=105,504)
Sociodemographic factors		
Age in years, median [IQR]	73 [65-79]	70 [62-77]
Women, n (%)	8,392 (72.6)	76,286 (72.3)
Index of multiple deprivation in quintiles, n (%)		
1 (least deprived)	2,328 (20.2)	21,062 (20.0)
5 (most deprived)	2,265 (19.6)	20,763 (19.8)
Ethnicity, n (%)		
White	7,982 (97.7)	62,766 (97.3)
Asian	100 (1.2)	768 (1.2)
Afro-Caribbean	30 (0.4)	448 (0.7)
Other	62 (0.8)	506 (0.8)
Duration of registration in years, median [IQR]	14.2 [6.6-26.8]	13.4 [6.0-25.3]
Consultation rate in previous year	10 [6-15]	5 [2-9]
Autoimmune diseases and inflammation		
Other autoimmune disease, n (%)	1,350 (11.7)	7,116 (6.7)
Cardiovascular risk factors		
Smoking, n (%)		
Current	1,023 (9.8)	10,397 (11.4)
Former	2,818 (27.0)	22,459 (24.6)
Never	6,587 (63.2)	58,584 (64.1)
Diabetes	777 (6.7)	6,526 (6.2)
Systolic blood pressure in mmHg, mean (SD)	144 (18.5)	143 (19.0)
Body mass index in kg/m^2, mean (SD)	26.8 (5.1)	26.7 (5.1)
Total cholesterol in mmol/L, mean (SD)	5.6 (1.2)	5.6 (1.1)
HDL cholesterol in mmol/L, mean (SD)	1.6 (0.5)	1.5 (0.5)
Serum creatinine in mg/L, mean (SD)	87.4 (23.9)	88.2 (24.3)
Medication use in previous year		
Immunosuppressant drugs, n (%)	156 (1.4)	593 (0.6)
Nonsteroidal anti-inflammatory drugs, n (%)	3,467 (30.0)	14,240 (13.5)
Oral corticosteroids, n (%)	5,566 (48.1)	2,476 (2.4)
Antiplatelet therapy, n (%)	1,013 (8.8)	8,787 (8.3)
Blood pressure lowering medication, n (%)	4,977 (43.0)	38,704 (36.7)
Statins, n (%)	1,117 (9.7)	9,230 (8.8)

Note: GCA, giant cell arteritis; HDL, high-density lipoprotein; IQR, interquartile range; PMR, polymyalgia rheumatica; SD, standard deviation

*Missing data (%): index of multiple deprivation, 0.4%; ethnic group, 37.9%; BMI, 41.8%; systolic blood pressure, 13.0%; smoking, 13.0%; total cholesterol, 61.2%; HDL cholesterol, 71.0%; serum creatinine, 47.6%.

Table S6. Crude incidence rates of twelve cardiovascular diseases with 95% confidence intervals in individuals with and without polymyalgia rheumatica and giant cell arteritis

	Patients with PMR and/or GCA (N=11,567)		Patients with PMR only (N=9,776)		Patients with GCA only (N=1,164)	
	No. events	Rate / 1000 PY (95% CI)	No. events	Rate / 1000 PY (95% CI)	No. events	Rate / 1000 PY (95% CI)
Stable angina	457	8.56 (7.81-9.38)	374	8.33 (7.53-9.22)	56	11.52 (8.86-14.97)
Unstable angina	101	1.89 (1.56-2.30)	89	1.98 (1.61-2.44)	7	1.44 (0.69-3.02)
Myocardial infarction	308	5.77 (5.16-6.45)	253	5.64 (4.98-6.38)	39	8.02 (5.86-10.98)
Unheralded coronary death	170	3.18 (2.74-3.70)	148	3.30 (2.81-3.87)	15	3.09 (1.86-5.12)
Cardiac arrest	73	1.37 (1.09-1.72)	64	1.43 (1.12-1.82)	2	0.41 (0.10-1.64)
Heart failure	759	14.21 (13.23-15.26)	619	13.79 (12.75-14.92)	94	19.33 (15.80-23.67)
Transient ischaemic attack	273	5.11 (4.54-5.75)	216	4.81 (4.21-5.50)	34	6.99 (5.00-9.79)
Ischaemic stroke	175	3.28 (2.82-3.80)	137	3.05 (2.58-3.61)	30	6.17 (4.31-8.83)
Subarachnoid haemorrhage	12	0.22 (0.13-0.40)	9	0.20 (0.10-0.39)	2	0.41 (0.10-1.64)
Intracerebral haemorrhage	54	1.01 (0.77-1.32)	42	0.94 (0.69-1.27)	8	1.65 (0.82-3.29)
Peripheral arterial disease	297	5.56 (4.96-6.23)	234	5.21 (4.59-5.93)	50	10.28 (7.79-13.57)
Abdominal aortic aneurysm	108	2.02 (1.67-2.44)	87	1.94 (1.57-2.39)	11	2.26 (1.25-4.09)

Note: CI, confidence interval; GCA, giant cell arteritis; PMR, polymyalgia rheumatica; Non-disease estimates are obtained among up to 10 randomly selected patients without polymyalgia rheumatica or giant cell arteritis matched for sex, age, medical practice and index date; PY, person-years of follow-up; Rates are unadjusted; Polymyalgia rheumatica and giant cell arteritis estimates are obtained among patients diagnosed with these diseases who had supporting information for disease diagnosis.

Table S7. Crude incidence rate of twelve cardiovascular diseases with 95% confidence intervals in individuals with incident and prevalent polymyalgia rheumatica and/or giant cell arteritis

	Patients without PMR or GCA		Patients with incident PMR/GCA		Patients with prevalent PMR/GCA	
	No.	Rate / 1000 PY	No.	Rate / 1000 PY	No.	Rate / 1000 PY
Stable angina	4006	9.55 (9.26-9.85)	258	7.53 (6.66-8.51)	199	10.39 (9.05-11.94)
Unstable angina	779	1.86 (1.73-1.99)	62	1.81 (1.41-2.32)	39	2.04 (1.49-2.79)
Myocardial infarction	2112	5.03 (4.82-5.25)	203	5.92 (5.16-6.80)	105	5.48 (4.53-6.64)
Unheralded coronary death	1516	3.61(3.44-3.80)	83	2.42 (1.95-3.00)	87	4.54 (3.68-5.61)
Cardiac arrest	511	1.22 (1.12-1.33)	44	1.28 (0.96-1.73)	29	1.51 (1.05-2.18)
Heart failure	5485	13.07 (12.73-13.42)	423	12.34 (11.22-13.58)	336	17.55 (15.77-19.53)
Transient ischaemic attack	2356	5.61 (5.39-5.85)	166	4.84 (4.16-5.64)	107	5.59 (4.62-6.75)
Ischaemic stroke	1425	3.40 (3.22-3.58)	114	3.33 (2.77-4.00)	61	3.19 (2.48-4.09)
Subarachnoid haemorrhage	139	0.33 (0.28-0.39)	9	0.26 (0.14-0.50)	3	0.16 (0.05-0.49)
Intracerebral haemorrhage	447	1.07 (0.97-1.17)	27	0.79 (0.54-1.15)	27	1.41 (0.97-2.06)
Peripheral arterial disease	2107	5.02 (4.81-5.24)	152	4.44 (3.78-5.20)	145	7.57 (6.44-8.91)
Abdominal aortic aneurysm	676	1.61 (1.49-1.74)	58	1.69 (1.31-2.19)	50	2.61 (1.98-3.45)
Composite endpoints						
Coronary and CVD death	10,826	25.80 (25.32-26.29)	763	22.26 (20.74-23.90)	528	27.58 (25.32-30.03)
Fatal and non-fatal CVD	24,372	58.08 (57.36-58.82)	1,736	50.66 (48.33-53.10)	1,276	66.64 (63.08-70.40)

Note: CI, confidence interval; CVD, cardiovascular disease; GCA, giant cell arteritis; PMR, polymyalgia rheumatica; Non-disease estimates are obtained among up to 10 randomly selected patients without polymyalgia rheumatica and giant cell arteritis matched for sex, age, medical practice and index date; PY, person-years of follow-up; Rates are unadjusted; Incident estimates are obtained among patients diagnosed with polymyalgia rheumatica or giant cell arteritis who had supporting information for disease diagnosis and were diagnosed with these diseases at study entry; Prevalent estimates are obtained among patients diagnosed with polymyalgia rheumatica or giant cell arteritis who had supporting information for disease diagnosis and had been diagnosed with these diseases before study entryThe coronary and CVD death composite endpoint includes: stable angina, myocardial infarction, coronary heart diseases not otherwise specified and any cardiovascular death. The fatal and non-fatal CVD composite endpoint additionally includes: transient ischemic attack, peripheral arterial disease, and non-fatal heart failure, ischemic or haemorrhagic stroke.

Table S8. Adjusted incidence rate ratios for the association between pure and concomitant PMR and GCA, and the initial presentation of cardiovascular diseases

	Patients with incident PMR and/or GCA IRR (95%CI)	Patients with prevalent PMR and/or GCA IRR (95%CI)	Patients with PMR only IRR (95%CI)	Patients with GCA only IRR (95%CI)
Coronary and CVD death composite				
Adjusted for age and sex	0.82 (0.76-0.88)	0.96 (0.88-1.05)	0.86 (0.81-0.91)	1.14 (0.99-1.31)
+ CVD risk factors	0.88 (0.79-0.98)	0.95 (0.76-1.18)	0.91 (0.83-1.00)	1.13 (0.61-2.09)
+ other autoimmune disease	0.88 (0.79-0.98)	0.94 (0.75-1.16)	0.91 (0.83-0.99)	1.13 (0.61-2.09)
+ Exclusion of patients				
with other autoimmune disease	0.89 (0.79-1.00)	0.92 (0.71-1.19)	0.91 (0.83-1.00)	1.13 (0.61-2.09)
≤6 months of follow-up	0.92 (0.82-1.02)	0.99 (0.78-1.26)	0.91 (0.83-1.00)	1.13 (0.61-2.09)
<50 years of age	0.85 (0.78-0.91)	0.99 (0.86-1.14)	0.88 (0.83-0.94)	1.21 (0.78-1.88)
+ Exclusion of 2yr unexposed follow-up for mixed cases*	0.88 (0.79-0.99)	0.91 (0.71-1.18)	0.90 (0.82-0.99)	1.12 (0.60-2.07)
+ Inclusion of patients with & without SID	0.88 (0.79-0.98)	0.93 (0.75-1.16)	0.91 (0.83-1.00)	1.13 (0.61-2.10)
+ Inclusion of first presentation of CVD**	0.87 (0.79-0.95)	1.03 (0.86-1.22)	0.91 (0.84-0.99)	1.13 (0.68-1.89)
Fatal and non-fatal CVD composite				
Adjusted for age and sex	0.82 (0.78-0.86)	0.99 (0.94-1.05)	0.86 (0.83-0.90)	1.26 (1.16-1.38)
+ CVD risk factors	0.85 (0.79-0.91)	0.99 (0.87-1.14)	0.88 (0.83-0.94)	1.21 (0.78-1.87)
+ other autoimmune disease	0.85 (0.79-0.91)	0.98 (0.85-1.12)	0.88 (0.83-0.94)	1.21 (0.78-1.87)
+ Exclusion of patients				
with other autoimmune disease	0.85 (0.78-0.92)	1.02 (0.88-1.19)	0.88 (0.83-0.94)	1.21 (0.78-1.87)
≤6 months of follow-up	0.85 (0.79-0.92)	1.03 (0.90-1.18)	0.88 (0.83-0.94)	1.21 (0.78-1.87)
<50 years of age	0.88 (0.79-0.98)	0.94 (0.75-1.18)	0.90 (0.82-0.99)	1.13 (0.61-2.10)
+ Exclusion of 2yr unexposed follow-up for mixed cases*	0.84 (0.78-0.91)	1.01 (0.87-1.18)	0.88 (0.82-0.93)	1.20 (0.77-1.85)
+ Inclusion of patients with & without SID	0.85 (0.79-0.91)	0.99 (0.86-1.13)	0.88 (0.83-0.94)	1.21 (0.78-1.87)
+ Inclusion of first presentation of CVD**	0.85 (0.79-0.91)	1.00 (0.87-1.15)	0.89 (0.83-0.94)	1.25 (0.83-1.90)

Note: CI, confidence intervals; CVD, cardiovascular disease; GCA, giant cell arteritis; IRR, incidence ratios; PMR, polymyalgia rheumatica; risk factors, cardiovascular disease risk factors included index of multiple deprivation, smoking status, systolic blood pressure, total and high-density lipoprotein cholesterol, body mass index and diabetes; SID, supporting information of disease diagnosis. The coronary and CVD death composite endpoint includes: stable angina, myocardial infarction, coronary heart

diseases not otherwise specified and any cardiovascular death. The fatal and non-fatal CVD composite endpoint additionally includes: transient ischemic attack, peripheral arterial disease, and non-fatal heart failure, ischemic or haemorrhagic stroke.

* Censoring of follow-up two years before diagnosis of PMR/GCA for patients who contributed to PMR/GCA and non-PMR/GCA analysis groups (i.e. patients with incident disease).

** Endpoints were the first presentation of the specific cardiovascular disease type regardless of prior occurrence of another type of cardiovascular disease

Table S9. Adjusted incidence rate ratios for the association between anti-inflammatory medication use and the initial presentation of twelve cardiovascular diseases

	NSAIDS		Immunosuppressant drugs	
	All patients	Patients with PMR and/or GCA	All patients	Patients with PMR and/or GCA
	IRR (95%CI)	IRR (95%CI)	IRR (95%CI)	IRR (95%CI)
Cardiac diseases				
Stable angina	0.84 (0.72-0.97)	0.85 (0.59-1.22)	0.86 (0.74-1.00)	0.83 (0.58-1.20)
Unstable angina	0.83 (0.59-1.17)	0.55 (0.34-0.92)	0.87 (0.70-1.21)	0.56 (0.34-0.93)
Myocardial infarction	1.14 (0.93-1.39)	0.59 (0.44-0.80)	1.15 (0.43-0.59)	0.59 (0.44-0.80)
Unheralded coronary death	0.76 (0.63-0.92)	0.56 (0.37-0.84)	0.78 (0.65-0.94)	0.55 (0.37-0.82)
Heart failure	0.97 (0.84-1.12)	1.09 (0.76-1.56)	0.98 (0.85-1.14)	1.08 (0.75-1.55)
Cardiac arrest	1.04 (0.64-1.68)	1.06 (0.58-1.92)	1.02 (0.63-1.65)	1.06 (0.58-1.92)
Cerebrovascular diseases				
Transient ischaemic attack	0.66 (0.53-0.82)	0.63 (0.47-0.85)	0.67 (0.54-0.84)	0.63 (0.47-0.86)
Ischaemic stroke	0.82 (0.63-1.07)	1.16 (0.76-1.79)	0.80 (0.62-1.04)	1.16 (0.75-1.78)
Subarachnoid haemorrhage	0.23 (0.03-1.69)	-	0.28 (0.04-2.14)	-
Intracerebral haemorrhage	0.92 (0.66-1.28)	0.66 (0.34-1.29)	0.92 (0.66-1.28)	0.67 (0.34-1.30)
Peripheral vascular diseases				
Peripheral arterial disease	0.97 (0.77-1.22)	0.91 (0.57-1.47)	1.02 (0.81-1.28)	0.92 (0.57-1.48)
Abdominal aortic aneurysm	1.26 (0.91-1.75)	0.41 (0.26-0.67)	1.24 (0.89-1.73)	0.42 (0.26-0.67)
Composite endpoints				
Coronary and CVD death	0.87 (0.79-0.96)	0.74 (0.65-0.85)	0.89 (0.81-0.98)	0.74 (0.65-0.85)
Fatal and non-fatal CVD	0.86 (0.811-0.92)	0.81 (0.74-0.89)	0.88 (0.82-0.94)	0.81 (0.74-0.89)

Note: CI, confidence interval; GCA, giant cell arteritis; IRR, incidence rate ratios adjusted for sex, age, polymyalgia rheumatica and giant cell arteritis diagnosis, index of multiple deprivation, smoking status, systolic blood pressure, total and high-density lipoprotein cholesterol, body mass index and diabetes; NSAIDS, non-steroidal anti-inflammatory drugs. Because of the limited number of events in specific clusters, estimates for unheralded coronary death and intracerebral haemorrhage were not adjusted for body mass index, systolic blood pressure, total and high-density lipoprotein cholesterol; and no estimates were computed for subarachnoid haemorrhage within the group of patients with polymyalgia rheumatica and giant cell arteritis. The coronary and CVD death composite endpoint includes: stable angina, myocardial infarction, coronary

heart diseases not otherwise specified and any cardiovascular death. The fatal and non-fatal CVD composite endpoint additionally includes: transient ischemic attack, peripheral arterial disease, and non-fatal heart failure, ischemic or haemorrhagic stroke.

Table S10. Adjusted incidence rate ratios for the association between pure and concomitant PMR and GCA, and the initial presentation of cardiovascular diseases, restricted to patients who had evidence of active disease

	**All PMR/GCA patients**	**PMR/GCA patients with active disease**
**Coronary & death composite**		
PMR only	0.91 (0.83-1.00)	0.90 (0.80-1.01)
GCA only	1.13 (0.61-2.09)	1.30 (0.59-2.88)
All GCA	0.67 (0.42-1.07)	0.77 (0.44-1.36)
PMR and/or GCA	0.90 (0.82-0.98)	0.89 (0.80-1.00)
**Fatal & non-fatal CVD composite**		
PMR only	0.88 (0.83-0.94)	0.86 (0.80-0.93)
GCA only	1.21 (0.78-1.87)	1.41 (0.83-2.41)
All GCA	0.80 (0.61-1.04)	0.84 (0.59-1.20)
PMR and/or GCA	0.88 (0.83-0.94)	0.86 (0.80-0.93)
**Stable angina**	0.86 (0.74-1.01)	0.79 (0.66-0.95)
**Unstable angina**	0.86 (0.62-1.20)	0.75 (0.50-1.13)
**Myocardial infarction**	1.16 (0.95-1.40)	1.33 (1.07-1.64)
**Unheralded coronary death**	0.79 (0.66-0.95)	0.66 (0.50-0.88)
**Heart failure**	0.99 (0.85-1.14)	1.00 (0.85-1.17)
**Cardiac arrest**	1.03 (0.64-1.67)	1.28 (0.90-1.84)
**Transient ischaemic attack**	0.67 (0.54-0.84)	0.71 (0.54-0.94)
**Ischaemic stroke**	0.80 (0.62-1.04)	0.76 (0.54-1.06)
**Intracerebral haemorrhage**	0.92 (0.66-1.28)	0.72 (0.43-1.19)
**Peripheral arterial disease**	1.01 (0.81-1.72)	0.91 (0.69-1.21)
**Abdominal aortic aneurysm**	1.24 (0.89-1.72)	1.11 (0.75-1.65)

Note: Active disease was defined as presence of baseline CRP>3.0 mg/L and/or record of elevated or abnormal ESR; GCA, giant cell arteritis; PMR, polymyalgia rheumatica.

Table S11. Description of diagnosis codes for patients who were diagnosed with both polymyalgia rheumatica and giant cell arteritis (n=627)

	GCA	PMR
Median no. of codes per patient (IQR)	5 (3-10)	5 (3-10)
Median maximum duration between recorded codes in days	473 (129-1328)	1017 (302-2042)
Median maximum duration between recorded codes (in days) among patients who had ≥1 year follow-up after each of the initial disease diagnosis	536 (142-1406) (n=383)	1055 (336-2082) (n=426)
Median maximum duration between recorded codes (in days) among patients who had ≥2 year follow-up after each of the initial disease diagnosis	556 (142-1468) (n=349)	1097 (385-2084) (n=389)

Table S12. Adjusted incidence rate ratios for the association between pure and concomitant PMR and GCA, and the initial presentation of cardiovascular diseases, restricted to patients who had ≥1 diagnosis code recorded after 1 year of the initial diagnosis

	Coronary and death composite	Fatal & non-fatal CVD composite
**PMR only**		
All patients (n=9,776)	0.91 (0.83-1.00)	0.88 (0.83-0.94)
Including only patients who had ≥1 diagnosis codes recorded after 1 year of the initial diagnosis (n=4,142)	0.81 (0.69-0.95)	0.86 (0.77-0.95)
**GCA only**		
All patients (n=1,164)	1.13 (0.61-2.09)	1.21 (0.78-1.87)
Including only patients who had ≥1 diagnosis codes recorded after 1 year of the initial diagnosis (n=286)	0.55 (0.07-4.16)	0.96 (0.37-2.50)

FIGURES

Figure S1. Electronic health record phenotyping algorithm for polymyalgia rheumatica and giant cell arteritis

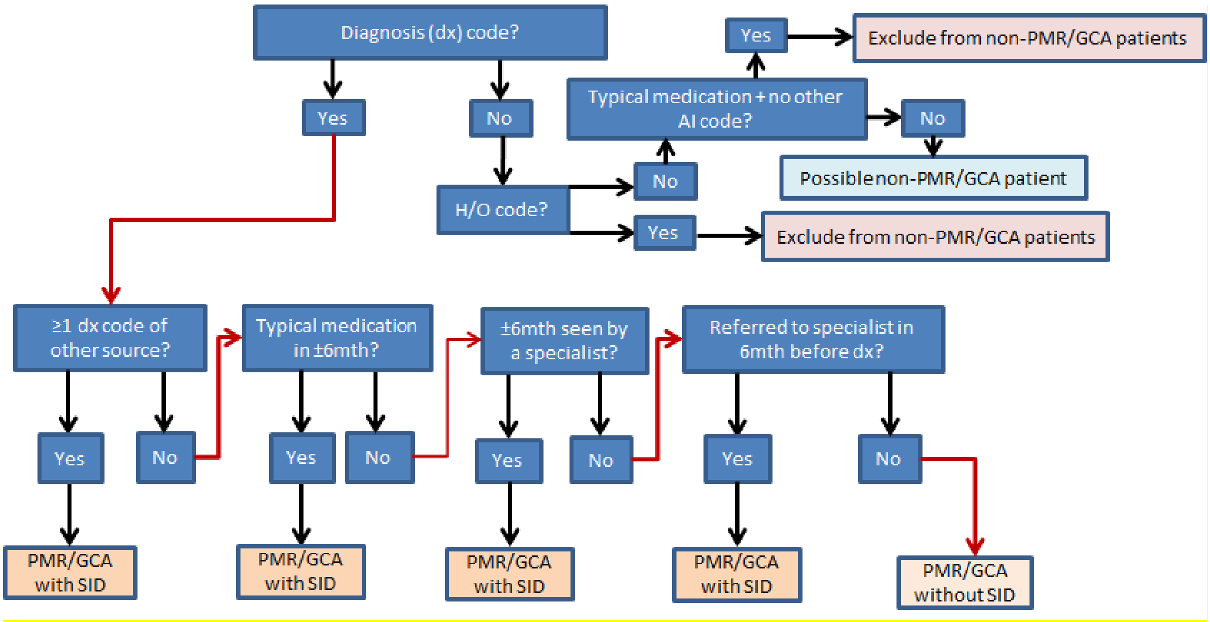

Note: AI, autoimmune disease; dx code, Read diagnosis code from Clinical Practice Research Datalink (CPRD, primary care) or ICD10 code from the Hospital Episode Statistics (HES, hospital); GCA, giant cell arteritis; H/O, history of; PMR, polymyalgia rheumatica; SID, supporting information for polymyalgia rheumatica or giant cell arteritis diagnosis. Primary analyses only included PMR/GCA patients with supporting information for diagnosis. In sensitivity analyses PMR/GCA patients with and without supporting information were included.

Figure S2. Polymyalgia rheumatica and giant cell arteritis and study definitions

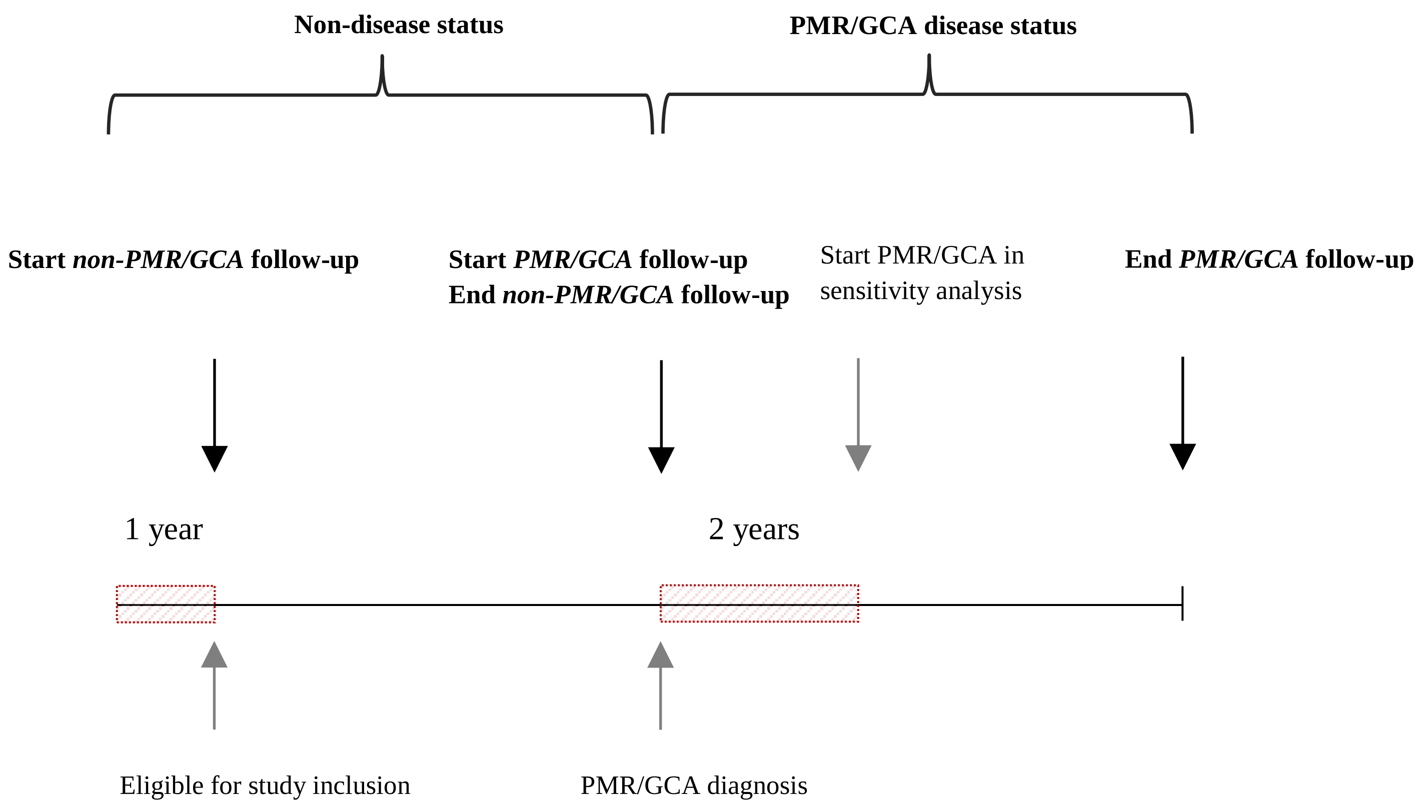

Note. GCA, giant cell arteritis; PMR, polymyalgia rheumatica; Patients with prevalent polymyalgia rheumatica or giant cell arteritis were those diagnosed prior to the date of eligibility for study inclusion. In combined PMR/GCA analyses, study follow-up started on the date of the first recorded diagnosis of the two diseases.

Figure S3. Sequential adjustment of incidence rate ratios for the association between polymyalgia rheumatica and/or giant cell arteritis, and the initial presentation of twelve cardiovascular diseases

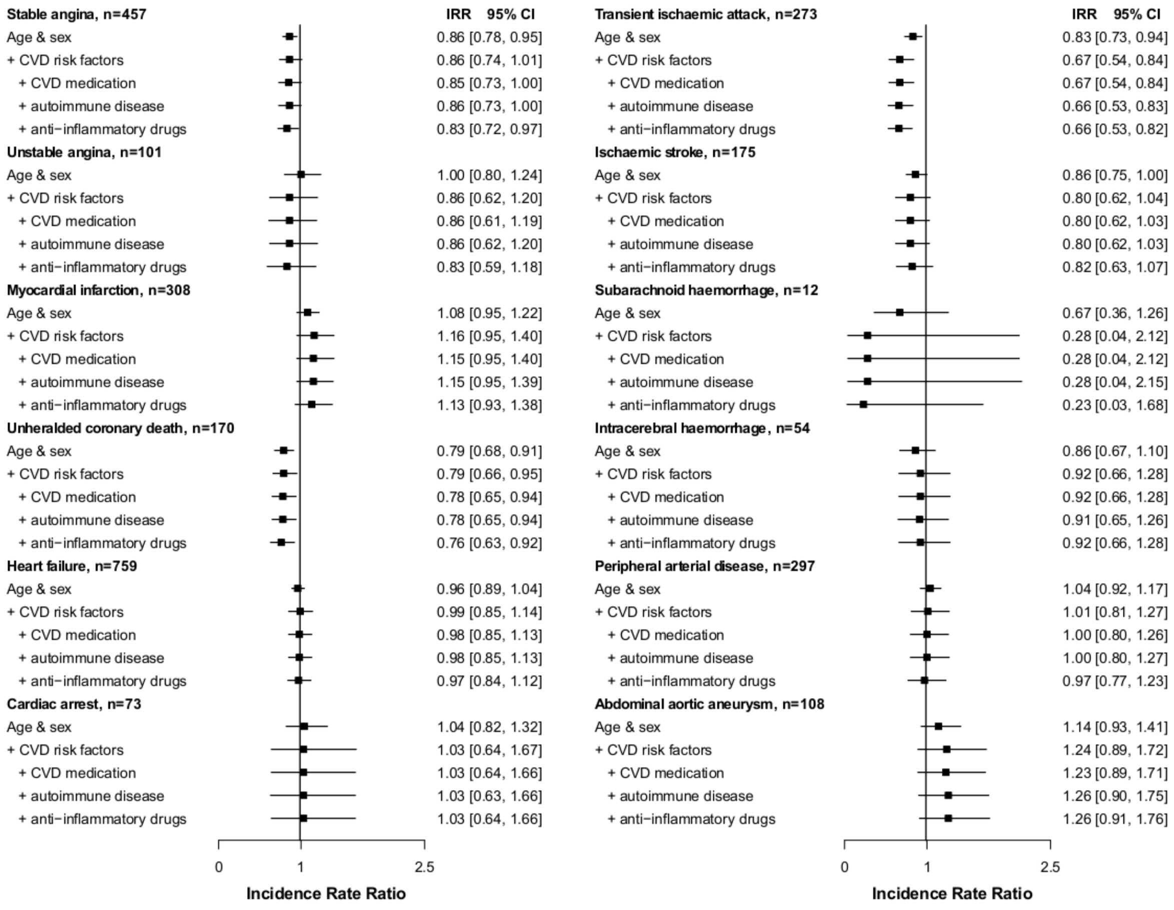

Note: CI, confidence interval; CVD, cardiovascular disease; CVD medications included use of blood pressure and lipid lowering medication; IRR, incidence rate ratios; n, number of events. CVD risk factors included index of multiple deprivation, smoking status, systolic blood pressure, total and high-density lipoprotein cholesterol, body mass index and diabetes. Because of the small number of events in specific clusters, estimates for unheralded coronary death and intracerebral haemorrhage were not adjusted for body mass index, systolic blood pressure, total and high-density lipoprotein cholesterol. Inflammatory medication included immunosuppressant drugs and nonsteroidal anti-inflammatory drugs.

Figure S4. Adjusted incidence rate ratios for the association between polymyalgia rheumatica and/or giant cell arteritis, and the initial presentation of twelve cardiovascular diseases, additionally adjusted for the effect of anti-inflammatory medication

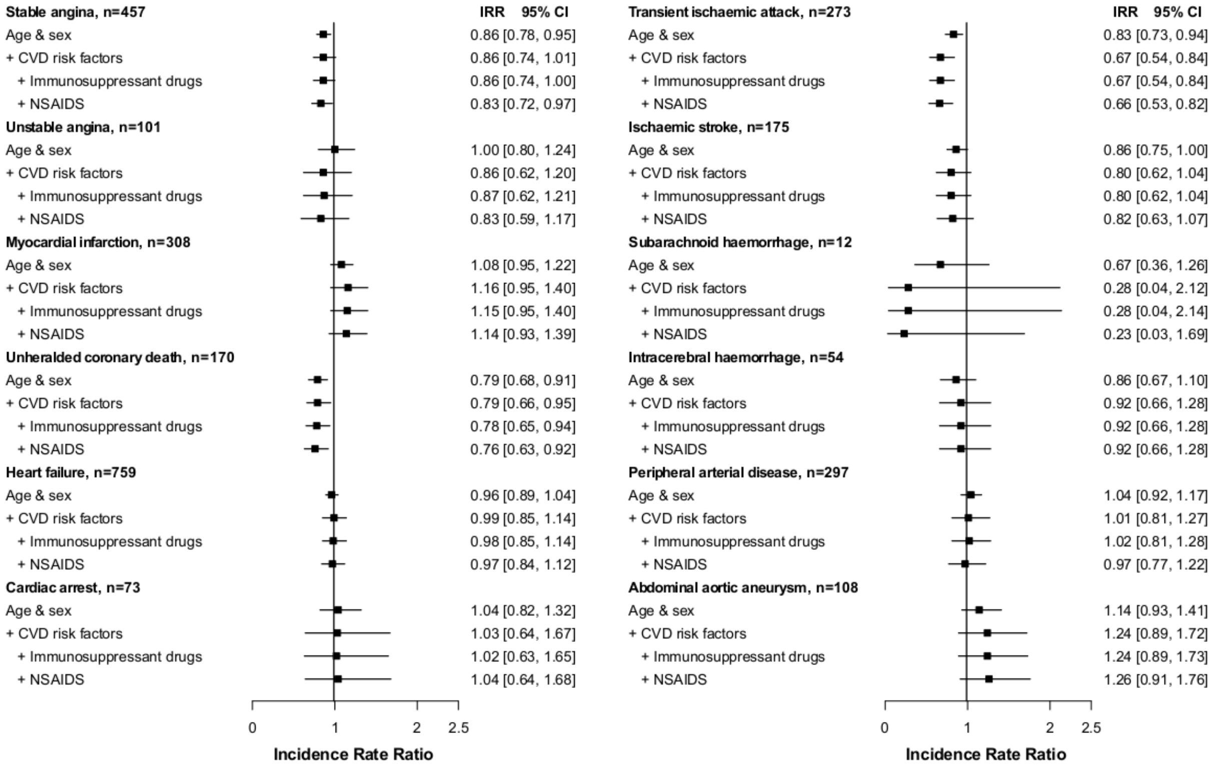

Note: CI, confidence interval; IRR, incidence rate ratios; CVD risk factors, cardiovascular risk factors included index of multiple deprivation, smoking status, systolic blood pressure, total and high-density lipoprotein cholesterol, body mass index and diabetes. Because of the small number of events in specific clusters, estimates for unheralded coronary death and intracerebral haemorrhage were not adjusted for body mass index, systolic blood pressure, total and high-density lipoprotein cholesterol; n, number of events; NSAIDS, non-steroidal anti-inflammatory drugs.

Figure S5. Adjusted incidence rate ratios for the association between polymyalgia rheumatica and/or giant cell arteritis, and the initial presentation of twelve cardiovascular diseases further adjusted for year of entry

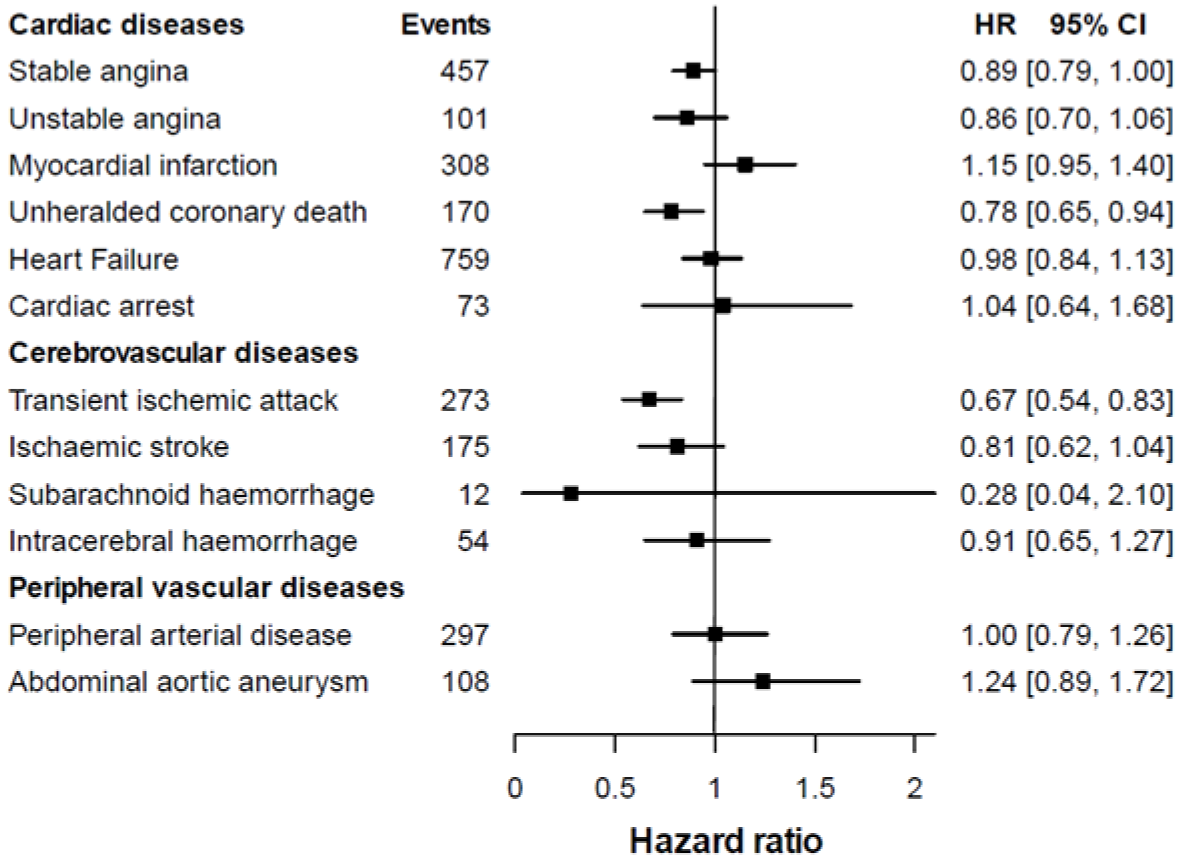

Note: CI, confidence interval; IRR, incidence rate ratios adjusted for sex, age, index of multiple deprivation, smoking status, systolic blood pressure, total and high-density lipoprotein cholesterol, body mass index and diabetes. Because of the small number of events in specific clusters, estimates for unheralded coronary death were not adjusted for body mass index, systolic blood pressure, total and high-density lipoprotein cholesterol.

Figure S6. Adjusted incidence rate ratios for the association between polymyalgia rheumatica and/or giant cell arteritis, and the initial presentation of twelve cardiovascular diseases stratified by age group

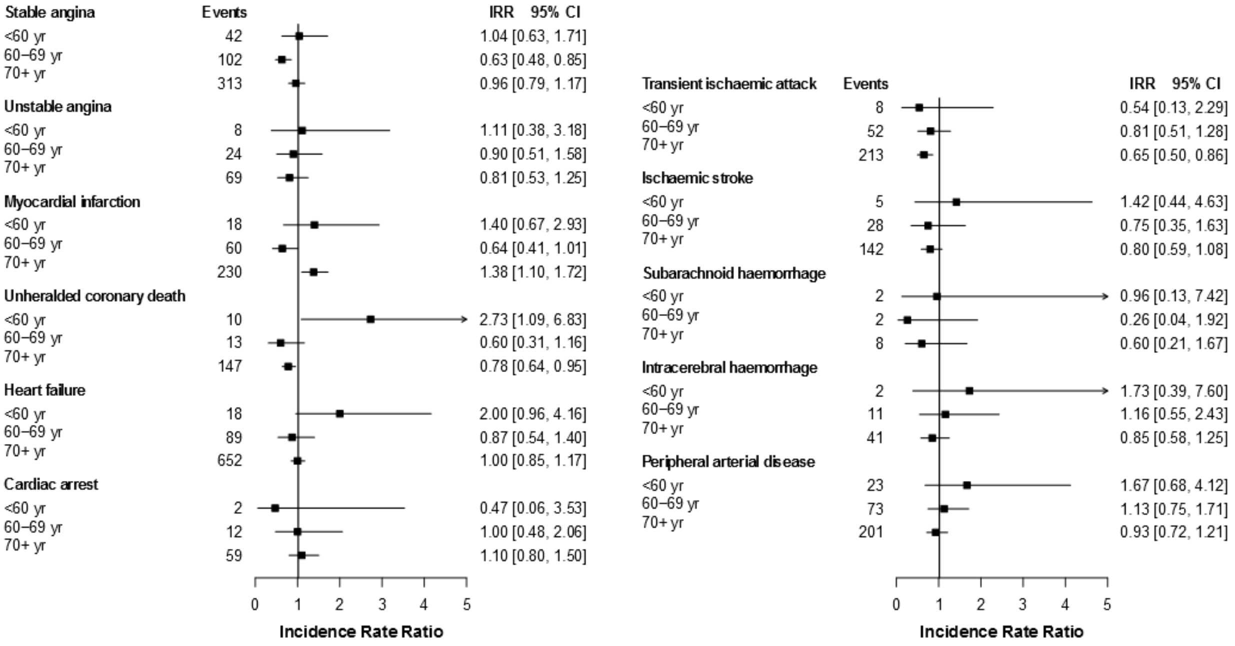

Note: CI, confidence interval; IRR, incidence rate ratios adjusted for sex, index of multiple deprivation, smoking status, systolic blood pressure, total and high-density lipoprotein cholesterol, body mass index and diabetes. Because of the small number of events in specific clusters, estimates for unheralded coronary death, intracerebral haemorrhage, subarachnoid haemorrhage and cardiac arrest are not adjusted for body mass index, systolic blood pressure, total and high-density lipoprotein cholesterol; and no estimates were obtained for abdominal aortic aneurysm.

Figure S7. Adjusted incidence rate ratios for the association between polymyalgia rheumatica and/or giant cell arteritis, and the initial presentation of twelve cardiovascular diseases in men and women

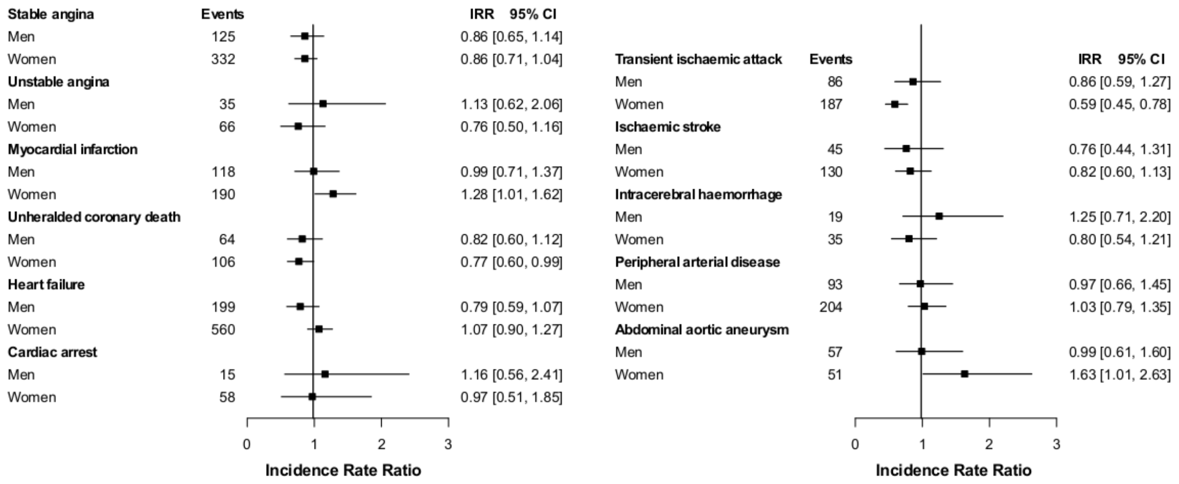

Note: CI, confidence interval; IRR, incidence rate ratios adjusted for age, index of multiple deprivation, smoking status, systolic blood pressure, total and high-density lipoprotein cholesterol, body mass index and diabetes. Because of the small number of events in specific clusters, estimates for unheralded coronary death and intracerebral haemorrhage were not adjusted for body mass index, systolic blood pressure, total and high-density lipoprotein cholesterol; and no estimates were obtained for subarachnoid haemorrhage.

Figure S8. Adjusted incidence rate ratios for the initial presentation of cardiovascular diseases stratified by disease duration (vs. <1 year duration) among patients with polymyalgia rheumatica and/or giant cell arteritis (vs. non-disease)

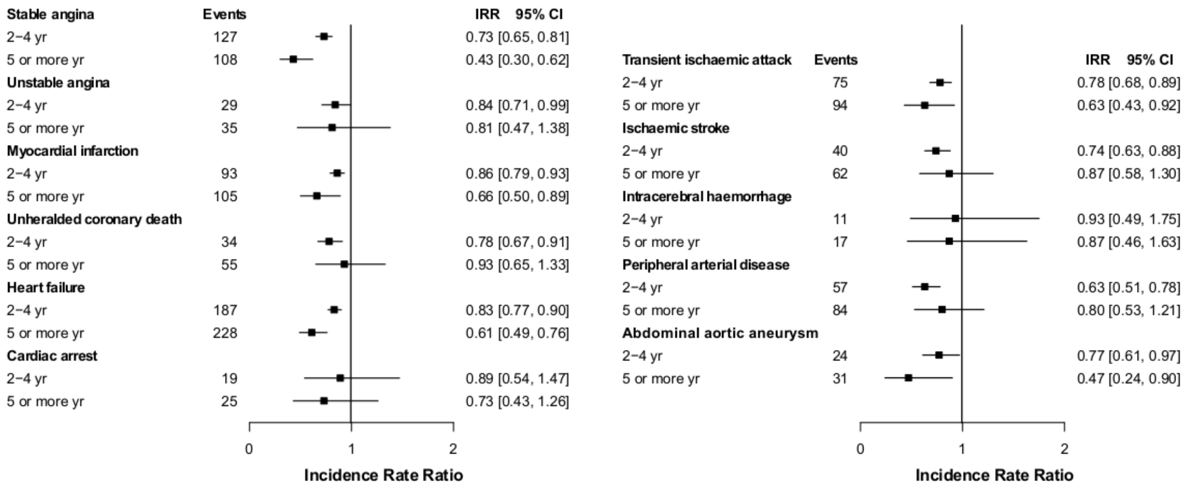

Note: CI, confidence interval; IRR, incidence rate ratios adjusted for index of age, sex, index of multiple deprivation, smoking status, systolic blood pressure, total and high-density lipoprotein cholesterol, body mass index and diabetes. Because of the small number of events in specific clusters, estimates for unheralded coronary death, cardiac arrest and intracerebral haemorrhage were not adjusted for body mass index, systolic blood pressure, total and high-density lipoprotein cholesterol; and no estimates were obtained for subarachnoid haemorrhage.

Figure S9. Adjusted incidence rate ratios of the initial presentation of twelve cardiovascular diseases for individuals with and without polymyalgia rheumatica, giant cell arteritis, or giant cell arteritis regardless of concomitant diagnosis with polymyalgia rheumatica (vs. no disease)

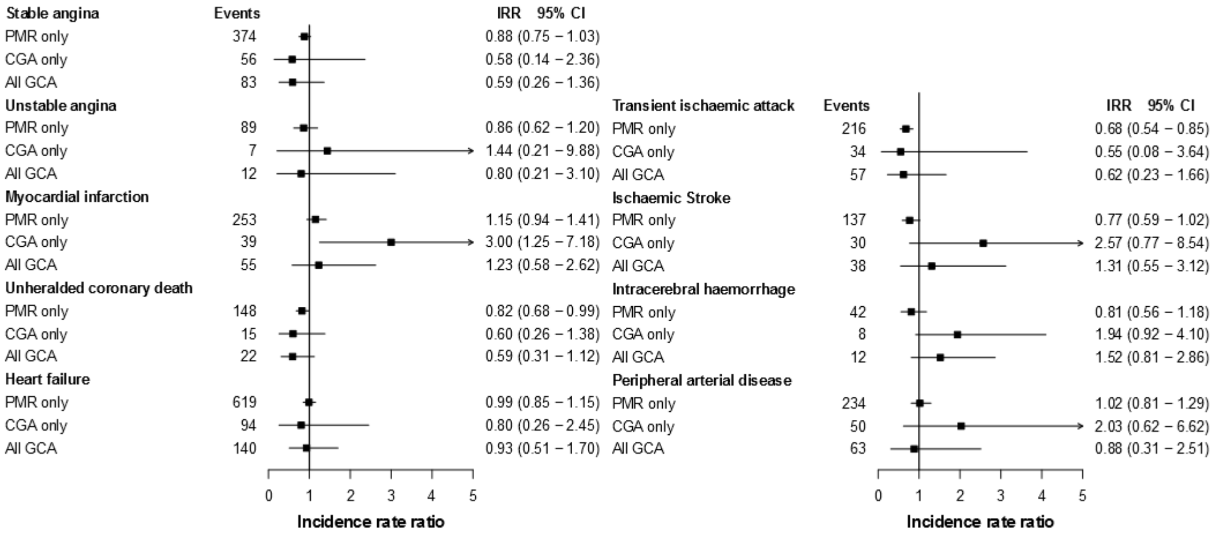

Note: CI, confidence interval; IRR, incidence rate ratios adjusted for sex, age, index of multiple deprivation, smoking status, systolic blood pressure, total and high-density lipoprotein cholesterol, body mass index and diabetes. Because of the small number of events in specific clusters, estimates for unheralded coronary death and intracerebral haemorrhage were not adjusted for body mass index, systolic blood pressure, total and high-density lipoprotein cholesterol. All CGA patients included patients only diagnosed with giant cell arteritis and patients with diagnosed both polymyalgia rheumatica and giant cell arteritis.

Figure S10. Adjusted incidence rate ratios for the association between polymyalgia rheumatica and/or giant cell arteritis, and the initial presentation of twelve cardiovascular diseases from sensitivity analyses.

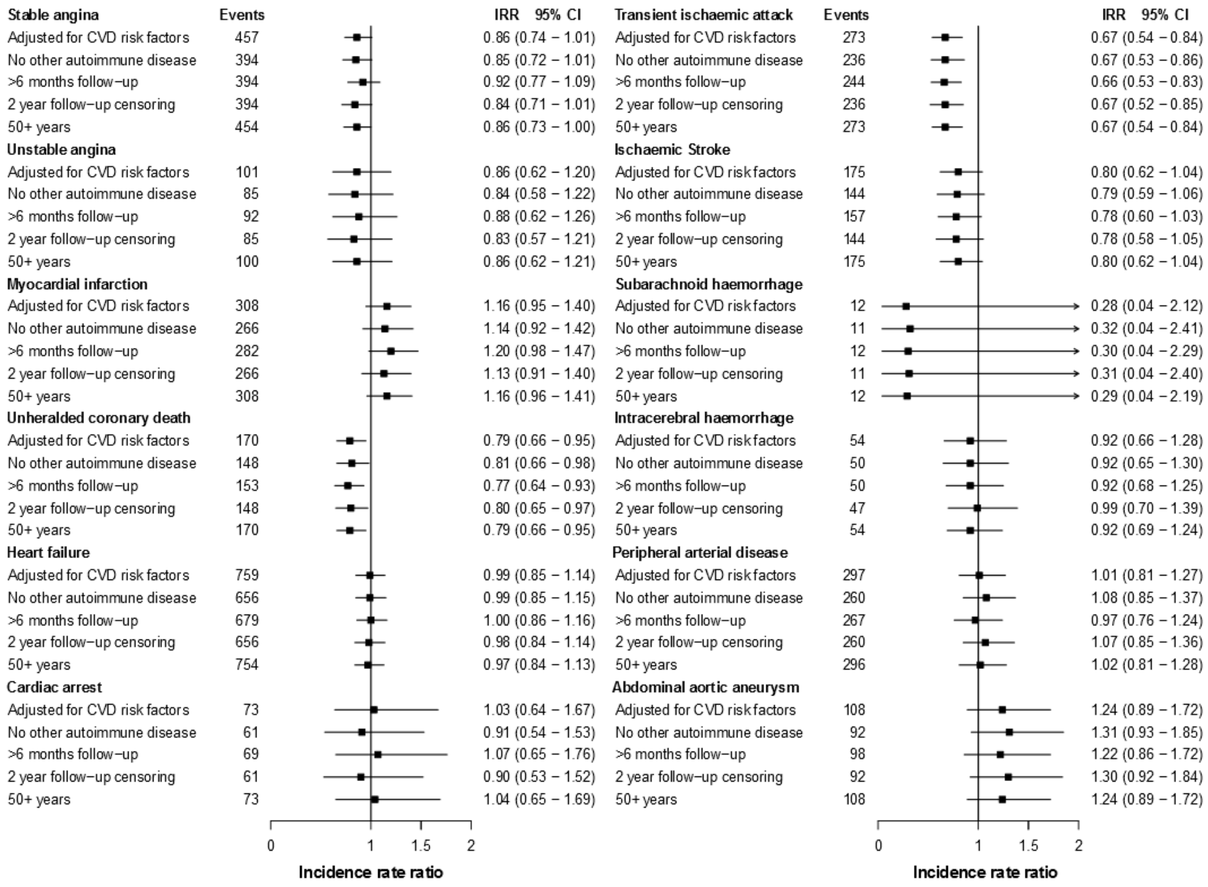

Note: CI, confidence interval; IRR, incidence rate ratios adjusted for sex, age, index of multiple deprivation, smoking status, systolic blood pressure, total and high-density lipoprotein cholesterol, body mass index and diabetes. Because of the small number of events in specific clusters, estimates for unheralded coronary death and intracerebral haemorrhage were not adjusted for body mass index, systolic blood pressure, total and high-density lipoprotein cholesterol. Sensitivity analyses were:

Second row (No other autoimmune disease): Exclusion of patients with a diagnosis of another autoimmune disorder.

Third row (>6 moths of follow-up): Exclusion of patients with less than 6 months of study follow-up.

Fourth row (2 year follow-up censoring): Exclusion of 2 years of study follow-up before the diagnosis of incident PMR/GCA for patients who contributed with follow-up to the PMR/GCA and non-PMR/GCA groups.

Fifth row (50+ years): Restriction of analysis to patients aged 50 years or older.

Figure S11. Comparison of adjusted incidence rate ratios for the association between polymyalgia rheumatica and/or giant cell arteritis, and the initial presentation of twelve cardiovascular diseases according to disease definition

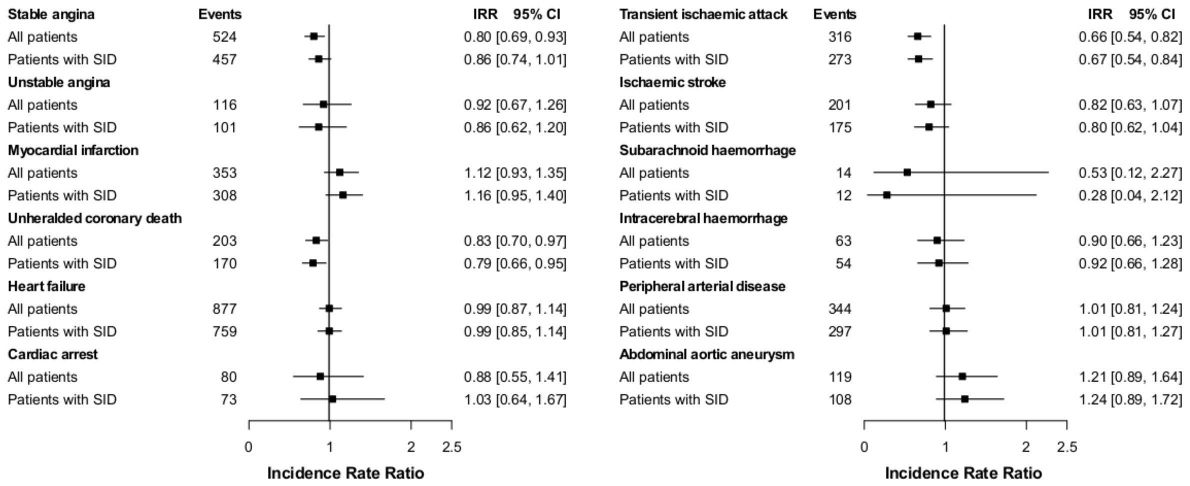

Note: CI, confidence interval; IRR, incidence rate ratios adjusted for sex, age, index of multiple deprivation, smoking status, systolic blood pressure, total and high-density lipoprotein cholesterol, body mass index and diabetes; SID, supporting information for diagnosis. Because of the small number of events in specific clusters, estimates for unheralded coronary death and intracerebral haemorrhage were not adjusted for body mass index, systolic blood pressure, total and high-density lipoprotein cholesterol. The SID population included patients with polymyalgia rheumatica and giant cell arteritis, who had supporting information of diagnosis and the corresponding up to 10 patients without disease matched for age, sex, medical practice and index date.

Figure S12. Adjusted incidence rate ratios for the association between polymyalgia rheumatica and/or giant cell arteritis, and the initial presentation of twelve cardiovascular diseases before and after the introduction of pay for performance (April 2004)

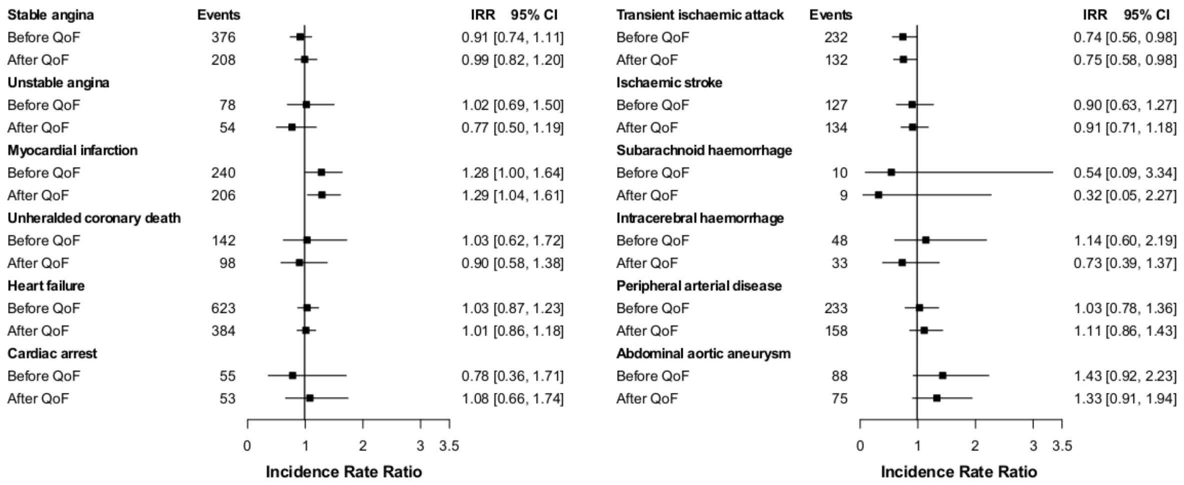

Note: CI, confidence interval; IRR, incidence rate ratios adjusted for sex, age, index of multiple deprivation, smoking status, systolic blood pressure, total and high-density lipoprotein cholesterol, body mass index and diabetes; QoF, Quality of Outcomes Framework. Because of the small number of events in specific clusters, estimates for unheralded coronary death and intracerebral haemorrhage were not adjusted for body mass index, systolic blood pressure, total and high-density lipoprotein cholesterol.

Figure S13. Adjusted incidence rate ratios for the association between polymyalgia rheumatica and/or giant cell arteritis, and the initial and first presentation of twelve cardiovascular diseases

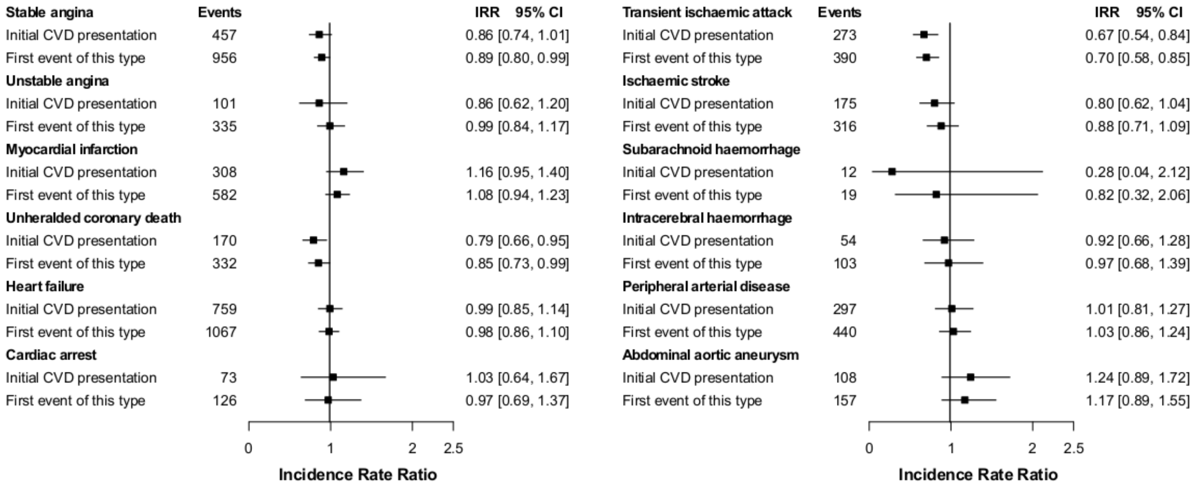

Note: CI, confidence interval; CVD, cardiovascular disease; IRR, incidence rate ratios adjusted for sex, age, index of multiple deprivation, smoking status, systolic blood pressure, total and high-density lipoprotein cholesterol, body mass index and diabetes. Because of the small number of events in specific clusters, estimates for unheralded coronary death were not adjusted for body mass index, systolic blood pressure, total and high-density lipoprotein cholesterol. The initial CVD presentation was the first presentation of any cardiovascular disease experienced by a patient (e.g. a myocardial infarction for a patient who experienced first a myocardial infarction and later developed heart failure). The first event of this type was the first presentation of cardiovascular disease for a patient regardless of prior occurrence of another type of cardiovascular disease (e.g. a patient who experienced first a myocardial infarction and later heart failure would contribute to both myocardial infarction and heart failure estimations).
